# Parvalbumin alters mitochondrial dynamics and affects cell morphology

**DOI:** 10.1007/s00018-018-2921-x

**Published:** 2018-09-25

**Authors:** Lucia Lichvarova, Thomas Henzi, Dzhamilja Safiulina, Allen Kaasik, Beat Schwaller

**Affiliations:** 1 Unit of Anatomy, Section of Medicine, University of Fribourg, Route Albert-Gockel 1, 1700 Fribourg, Switzerland; 2 Department of Pharmacology, Institute of Biomedicine and Translational Medicine, University of Tartu, 50411 Tartu, Estonia

**Keywords:** Parvalbumin, Mitochondria, Calcium signaling, Mitochondria dynamics, Homeostasis, Fusion–fission, Mitophagy

## Abstract

The Ca^2+^-binding protein parvalbumin (PV) and mitochondria play important roles in Ca^2+^ signaling, buffering and sequestration. Antagonistic regulation of PV and mitochondrial volume is observed in in vitro and in vivo model systems. Changes in mitochondrial morphology, mitochondrial volume and dynamics (fusion, fission, mitophagy) resulting from modulation of PV were investigated in MDCK epithelial cells with stable overexpression/downregulation of PV. Increased PV levels resulted in smaller, roundish cells and shorter mitochondria, the latter phenomenon related to reduced fusion rates and decreased expression of genes involved in mitochondrial fusion. PV-overexpressing cells displayed increased mitophagy, a likely cause for the decreased mitochondrial volumes and the smaller overall cell size. Cells showed lower mobility in vitro, paralleled by reduced protrusions. Constitutive PV down-regulation in PV-overexpressing cells reverted mitochondrial morphology and fractional volume to the state present in control MDCK cells, resulting from increased mitochondrial movement and augmented fusion rates. PV-modulated, bi-directional and reversible mitochondrial dynamics are key to regulation of mitochondrial volume.

**Electronic supplementary material:**

The online version of this article (10.1007/s00018-018-2921-x) contains supplementary material, which is available to authorized users.

## Introduction

Parvalbumin (PV) is a cytosolic Ca^2+^-binding protein of the large EF-hand protein family, implicated in intracellular Ca^2+^ regulation and trafficking [12, 75, 79]. PV is highly expressed in fast-twitch muscles and distinct neuron subpopulations, where PV plays an important role in Ca^2+^ signaling, e.g. by increasing the relaxation rate of fast-twitch muscles [78] or by modulation of short-term synaptic plasticity in PV-expressing neurons [11, 64]. It is also expressed in mouse renal epithelial cells of the distal convoluted tubule, where PV is suggested to function as an intracellular Ca^2+^ shuttle involved in transcellular Ca^2+^ resorption [41]. PV is considered as a slow-onset Ca^2+^ buffer and more precisely, as a Ca^2+^ signal modulator with two high-affinity Ca^2+^/Mg^2+^ mixed metal-binding sites [76, 79]. An inverse (antagonistic) regulation of PV expression and mitochondrial content has been confirmed by various approaches in several in vivo or in vitro model systems; for instance in fast-twitch muscles [16, 67] and Purkinje cells of PV knockout (PV−/−) mice [17], in striatal neurons of mice ectopically expressing PV (Thy-PV) [58], C2C12 myotubes [31] and Madin–Darby canine kidney (MDCK) epithelial cells [41]. Interestingly, alteration of PV expression levels does not affect levels of other co-expressed EF-hand Ca^2+^-binding proteins (e.g. calbindin D-28k in Purkinje cells of PV−/− mice [17]), but leads to significant changes in mitochondrial volume. Upregulation of PV in MDCK cells decreases transcript expression of prototypical mitochondrial genes, mostly ones with a function in mitochondrial Ca^2+^ transport and mitochondrial membrane potential including mitochondrial calcium uniporter (*Mcu*), mitochondrial calcium uniporter regulator1 (*Mcur1*), mitochondrial calcium uptake 1 (*Micu1*), uncoupling protein 2 (*Ucp2*), and mitocalcin (*Efhd1*) [41]. Also, the relative mitochondrial mass is decreased by 40–50% in MDCK cells with ectopic PV expression (PV-MDCK cells). Functionally, the collapse of the mitochondrial membrane potential by carbonyl cyanide *m*-chlorophenyl hydrazone (CCCP, mitochondrial oxidative phosphorylation uncoupler) occurs at lower concentrations in PV-MDCK cells compared to control MDCK (C-MDCK) cells [41]. All these evidences point out to a complex crosstalk between PV expression levels and mitochondrial volume and/or function. Mitochondrial volume density is regulated by mitochondrial fusion, fission and mitophagy, all processes implicated in and defined as mitochondrial dynamics, briefly summarized here.

Mitochondrial dynamics is critical for mitochondrial health and quality control and maintaining of mitochondrial homeostasis [13, 18, 51, 71]. Proteins responsible for mitochondrial fusion and fission events are relatively well characterized [13, 18] and include proteins of the inner (IMM) and outer mitochondrial membrane (OMM). Proteins localized in the OMM, Mitofusin 1 (*Mfn1*) and Mitofusin 2 (*Mfn2*) are necessary to link two separate mitochondria together and initiate the fusion of the OMM [70, 72], while the fusion of IMM is regulated by the dynamin-related GTPase OPA1 [30, 43]. For mitochondrial fission, the dynamin-related protein Drp1 is required [14, 80, 84]. As a large part of Drp1 is localized in the cytosol, different mitochondrial receptors and/or adaptors for Drp1 are indispensable to mediate squeezing and separation of mitochondria. Initially, Fis1 had been considered as a critical protein for mitochondrial fission [81, 92], but evidence has accumulated that Mff is an important adaptor protein for Drp1 and hence more relevant for the recruitment of Drp1 to mitochondrial membranes [38, 65]. Besides mechanisms regulating mitochondrial morphology by fusion–fission events, there is machinery to maintain a healthy mitochondrial population through mitochondrial biogenesis or by selective elimination of mitochondria by autophagy (mitophagy). The peroxisome proliferator-activated receptor γ coactivator-1 alpha (PGC-1α) is considered as a master regulator of mitochondrial biogenesis and cellular energy metabolism [90]. When mitochondria are damaged, they are generally eliminated by mitophagy [48, 53, 69]. Since smaller mitochondria are more suitable for autophagosomal encapsulation, it is assumed that mitochondrial fission is an important process to reduce mitochondrial size and subsequently mitochondrial volume [8, 53]. Selective elimination of mitochondria is primarily induced by the loss of the mitochondrial membrane potential; mitochondrial depolarization leads to an accumulation of the mitochondrial kinase PTEN-induced putative kinase 1 (PINK1) on the OMM of dysfunctional mitochondria [51, 52]. Subsequently ubiquitin ligase Parkin (PARK2) is recruited to mitochondria [51, 63]. PARK2 then ubiquitinates mitochondrial OMM proteins and tags mitochondria for degradation [46].

Here, we set out to investigate changes in mitochondrial dynamics (fusion, fission and mitophagy) caused by modulation of PV expression in MDCK cells serving as an easily accessible experimental model system. Based on our previous experiments demonstrating an inverse correlation/regulation between PV levels and mitochondrial volume in neurons [17], fast-twitch muscles [31], as well as in MDCK cells [41], we expect results obtained in MDCK cells to be translatable to the situation prevailing in neurons and fast-twitch muscle fibers with altered PV expression.

## Results

To further expand the mechanistic knowledge on the inverse regulation of PV and mitochondrial volume, we used MDCK cells and genetically modified MDCK cell lines, as described previously [41]. Besides the control (parental) PV-negative C-MDCK cells, we used cells ectopically expressing PV (PV-MDCK cells) and a third line, where PV expression in PV-MDCK cells was constitutively down-regulated by *Pvalb* shRNA (PV/shPV-MDCK cells). In these three lines, we had previously determined differentially expressed genes implicated in mitochondrial Ca^2+^ transport and membrane potential [41]. Here, MDCK cells were selected as a reliable model to evaluate modulation of mitochondrial dynamics by PV.

**Fig. 1:**
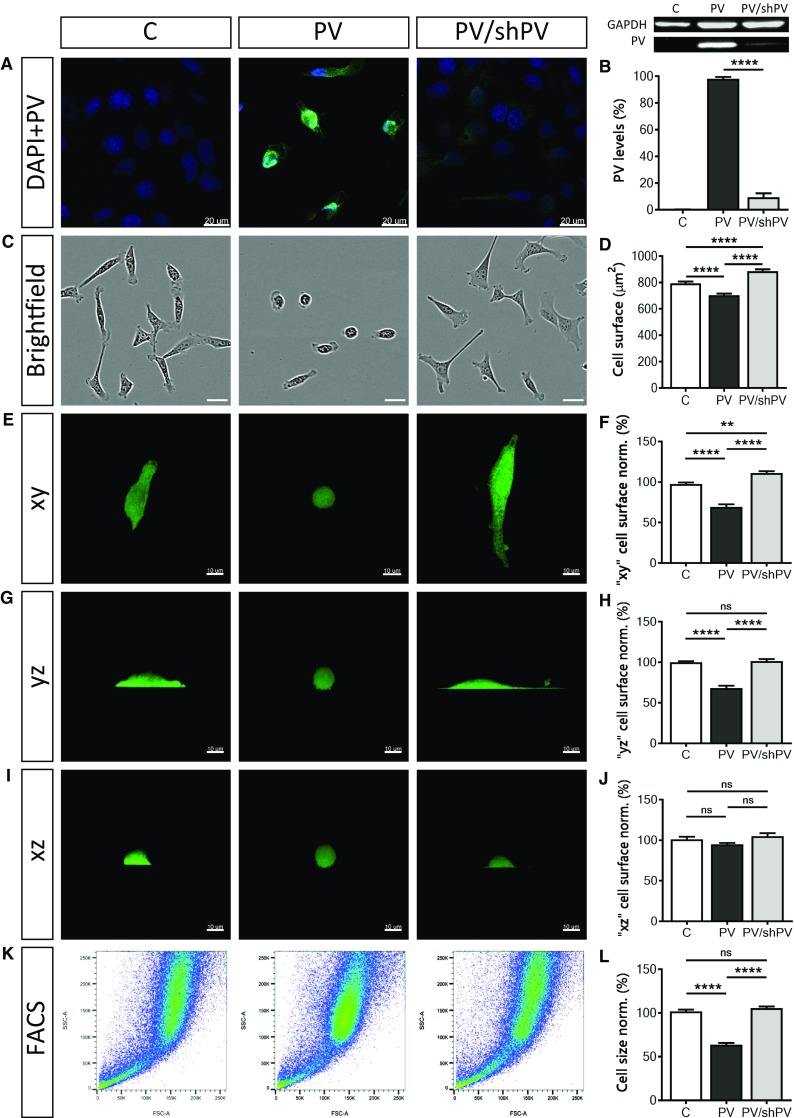
Parvalbumin overexpression decreases cell surface area and cell size. Detection of PV (green) and nuclear (blue) staining by immunocytochemistry (**a**). Signal for PV (green) was below the threshold for detection in control C-MDCK cells, yet nuclei were stained by DAPI (blue). Protein expression levels of PV in the three MDCK cell lines were determined also by Western blot analysis (**b**). For the normalization, GAPDH signals were used. Values were from three independent experiments. Representative images of MDCK cells acquired using the IncuCyte Live-Cell Imaging system (**c**). The surface area of more than 400 cells in each group was quantified (**d**). MDCK cells reconstructed in Imaris software were rotated to show their surface area at different angles—*xy* (**e**), *yz* (**g**) and *xz* (**i**) view. More than 200 cells were analyzed in each set (**f**, **h**, **j**). Representative graphs from flow cytometry analysis show forward scatter and side scatter (**k**) of 1 × 10^6^ MDCK cells. Analysis was done in FlowJo software and normalized to control cells (**l**)

**Fig. 2:**
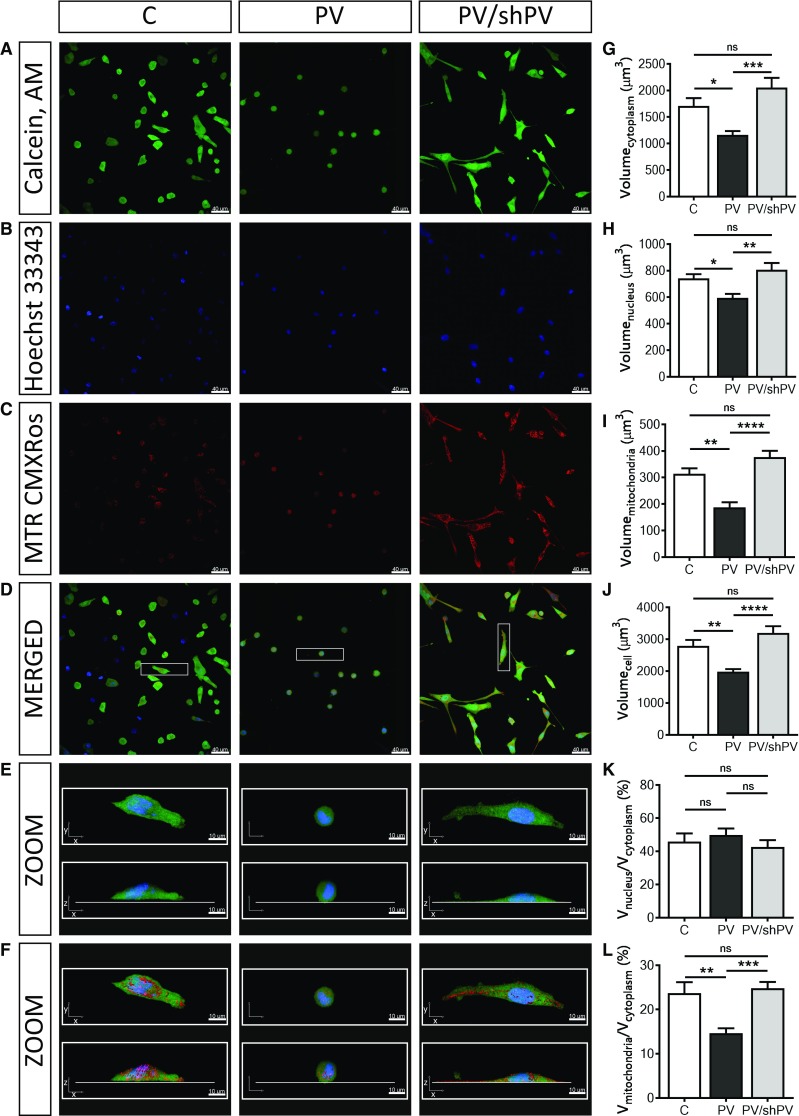
Parvalbumin decreases the whole-cell volume and the mitochondrial (absolute and relative) volume of MDCK cells. Representative confocal images showing the cytoplasm (**a**) in green (Calcein-AM), nuclei (**b**) in blue (Hoechst 33342), and mitochondria (**c**) in red (MitoTracker™ Red CMXRos). Selected representative cells (white rectangle) from merged images (**d**) are shown also at higher magnification (**e**) as *xy*- and *xz*-views of the same cells and in a 3D view with red-stained mitochondria also in *xy*- and *xz*-views (**f**). Individual cells were reconstructed in Imaris software analyzed in the “Cell mode” and “Surface mode”. More than 200 cells were analyzed per group from five independent experiments. Cells undergoing mitosis and cells at the edges of the microscope field of view were excluded from analysis. Stereological analysis of 3D-reconstructed MDCK cells revealed that the volume of the cytoplasm (**g**), nuclei (**h**), mitochondria (**i**) and of the whole cell (**j**) were significantly decreased in MDCK cells with ectopic expression of PV. There was also a significant decrease in the ratio of mitochondrial and cytoplasmic volumes (**l**) in the PV-overexpressing group, while the ratio of nuclei to cytoplasm (**k**) remained unchanged; see also supplementary movies showing representative MDCK cells

PV expression levels in the three MDCK cell lines were determined by immunocytochemistry (Fig. 1a) and by semi-quantitative Western blot analysis (Fig. 1b). In control C-MDCK cells, the expression level of PV was below the threshold for detection by either PV immunostaining or by Western blot analysis. The signal for GAPDH was used for the normalization of the PV signals (Fig. 1b). To compare relative PV expression levels, the signal of PV-MDCK cells was defined as 100%. Significantly lower PV levels (10.43 ± 0.88%) were detected in PV/shPV-MDCK cells. For the estimation of the PV concentration in PV-MDCK and PV/shPV-MDCK cells we first determined the amount of PV present in a MDCK cell (5.77 ± 0.88 ng) and in a PV/shPV-MDCK cell (0.78 ± 0.38 ng) using a calibration curve with purified PV (Suppl. Fig. S1). Based on the measurements of MDCK cell volumes (Fig. 2j), the intracellular PV concentration was calculated and found to be 480 µM in PV-MDCK and approximately 33 µM in PV/shPV-MDCK cells. The PV concentration observed in PV-MDCK cells falls well within the range of PV concentrations found in neurons (e.g. Purkinje cells or cerebellar interneurons) ranging from 50 to 1500 µM depending on cell type and species [22, 33, 75].

### PV decreases the cell surface area and attachment to the culture dishes

PV overexpression in MDCK cells resulted in smaller, roundish cells. In contrast, shPV/PV-MDCK cells were more flattened and appeared even bigger than C-MDCK cells. From more than 500 cells in 54 fields that were analyzed in each group, 16.67 ± 1.33% of cells with a round shape, with a circularity coefficient ≥ 0.8, were found in the C-MDCK group. Much more roundish cells (82.33 ± 3.25%) were detected in the PV-MDCK group (*P* < 0.0001), while in cells with downregulated PV (PV/shPV group) only 14.00 ± 2.00% were round shaped. Choosing a lower boundary (circularity coefficient ≥ 0.7 and ≥ 0.6) did not qualitatively change the results; the percentage of round cells was increased only in PV-MDCK cells (data not shown). To quantify the 2D cell surface area, brightfield images of MDCK cells were analyzed (Fig. 1c). The surface area (Fig. 1d) of PV-MDCK cells was smaller compared to C-MDCK cells (703.4 ± 12.2 vs. 794.1 ± 12.8 µm^2^, *P* < 0.0001) and compared to shPV/PV-MDCK cells (703.4 ± 14.2 vs. 885.4 ± 14.2 µm^2^, *P* < 0.0001). Note that the surface area of shPV/PV-MDCK cells was even larger than the surface area of C-MDCK cells (885.4 ± 14.2 vs. 794.1 ± 12.8 µm^2^, *P* < 0.0001). The surface area was also calculated from 3D-reconstructed confocal images (Fig. 1f, h, j). Representative individual cells are depicted as *xy* (Fig. 1e), *yz* (Fig. 1g) and *xz* (Fig. 1i) views. The surface areas calculated from *xy* views (Fig. 1e, f) were in agreement with the results from brightfield images (Fig. 1c, d), the surface area of PV-MDCK cells was smaller compared to either C-MDCK (30% decrease, *P < *0.0001) or shPV/PV-MDCK cells (40% decrease, *P* < 0.0001). In *xz* and *yz* views the zones of PV-MDCK cells in direct contact with the culture dishes were clearly smaller than in the other two MDCK lines with none-to-low PV expression, see lower (bottom) part of the cells (Fig. 1g, i). Control and shPV/PV-MDCK cells showed tight interaction with the collagen surface coating. The irregular and sparse interaction sites with the surface of the dishes are indicative of poor cell attachment of PV-MDCK cells (Fig. 1g, i, middle panel). Relative total cell size measurements by FACS (Fig. 1k, l) revealed PV-MDCK cells to be approximately half the size of C-MDCK and shPV/PV-MDCK cells (*P* < 0.0001 vs. C-MDCK and *P* < 0.0001 vs. shPV/PV-MDCK cells) and no significant differences between C-MDCK cells and shPV/PV-MDCK cells (*P* = 0.4086).

### Parvalbumin decreases the whole-cell volume, as well as volumes of the cytoplasm, mitochondria and nuclei: phenotype reversal in shPV/PV-MDCK cells

**Fig. 3:**
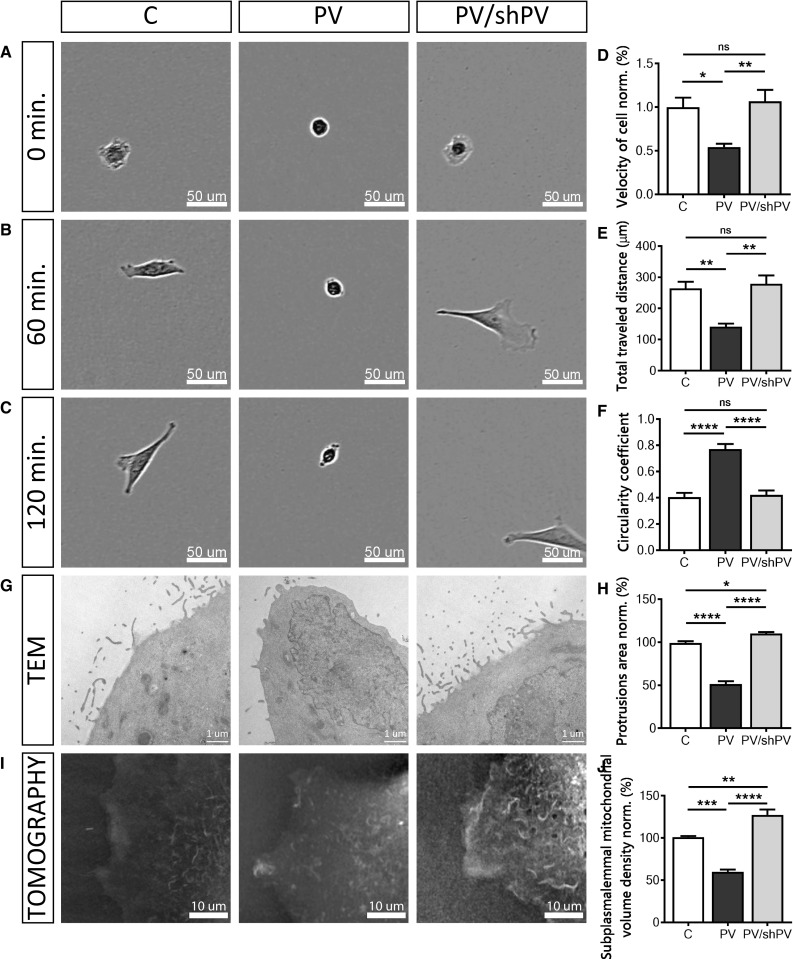
Parvalbumin decreases the cell motility. Brightfield images taken at 0 (**a**), 60 (**b**), and 120 min (**c**) after seeding of the three MDCK cell lines (C-MDCK, PV-MDCK, shPV/PV-MDCK). Measurements of relative velocity normalized to C-MDCK cells (**d**) and total distance travelled during 120 min (**e**). Evaluation of circularity coefficient of the MDCK cell lines. The quantification of at least 100 cells in each group is shown (**f**). Representative TEM images of protrusions (filopodia, lamellipodia) in MDCK cells (**g**) were taken at 24,500× magnification. The quantification of at least 15 cells in each group is shown (**h**). Representative images from tomography (**i**) showing mitochondria (light color) and plasma membrane based on the refraction index. Subplasmalemmal mitochondrial volume density normalized to C-MDCK cells was analyzed using STEVE software (**j**)

The relative mitochondrial mass of MDCK cells had been previously determined by FACS analysis using MitoTracker™ Green FM [41]. To gain more detailed information about the mitochondrial morphology in MDCK cells, Z-stack confocal microscopy imaging followed by 3D reconstruction was performed. Cells were stained with (1) Calcein-AM for determining the cytosolic compartment of viable cells (Fig. 2a), (2) Hoechst 33342 for nuclei (Fig. 2b) and (3) MitoTracker™ Red CMXRos for detecting functionally intact mitochondria (Fig. 2c). Merged images are shown in Fig. 2d and representative cells are further shown in ZOOM and reconstructed in 3D (Fig. 2e, f); animated views of representative cells are shown in Suppl. movies 1–3. More than 200 cells per cell line were analyzed, collected from five independent experiments. Cytoplasmic (Fig. 2g), nuclear (Fig. 2h) and mitochondrial (Fig. 2i) volumes were estimated by semi-quantitative volume measurements and ratios nucleus/cytoplasm (Fig. 2k) and mitochondria/cytoplasm volumes (Fig. 2l) were calculated. Not only the mitochondrial volume was decreased by 40% in PV-MDCK cells (187.4 ± 19.2 vs. 313.8 ± 20.5 µm^3^, *P* = 0.0013; Fig. 2c, i), but also whole-cell volumes were approximately 30% smaller than of C-MDCK cells (1982 ± 79 vs. 2789 ± 190 µm^3^, *P* = 0.0063, Fig. 2d, j). Of note, the decrease in mitochondrial volume alone cannot account for the substantial decrease in overall cell volume. The decrease in PV expression levels in shPV/PV-MDCK cells led to a restoration of whole-cell volumes to volumes of C-MDCK cells (3194 ± 214 vs. 2789 ± 190 µm^3^, *P* = 0.2399) and also mitochondrial volumes reverted to the state prevailing in C-MDCK cells (Fig. 2i). C-MDCK cells showed a typical mitochondrial distribution in the cytoplasm (Fig. 2f); tubular and globular mitochondria occupied 20–30% of the cytoplasmic area (Fig. 2l). In contrast, mitochondria in PV-MDCK cells were roundish, localized closer to the nucleus and the fractional mitochondrial volume (ratio of mitochondrial volume and volume of cytoplasm) was decreased (14.7 ± 1.1 in PV-MDCK cells compared to 23.7 ± 2.5% in C-MDCK cells, *P* = 0.0018). Long tubular mitochondria were observed in shPV/PV-MDCK cells, with mitochondria localized mostly in subplasmalemmal regions at the bottom of cells. Resulting from the flattening, shPV/PV-MDCK cells appeared even bigger than C-MDCK cells on 2D images (Figs. 1c, e, 2a, d–f). Nevertheless, no significant differences were observed between C-MDCK and shPV/PV-MDCK cells by 3D-volumetric analyses (Fig. 2g–l). Of importance, when comparing fractional mitochondrial volumes between PV-MDCK and shPV/PV-MDCK cells, the decreased PV levels led to a significant increase in mitochondrial volume to 24.8 ± 1.5% in shPV/PV-MDCK cells, *P* = 0.0003 vs. PV-MDCK) and reaching values similar to C-MDCK cells (*P* = 0.8936). Unexpectedly, also volumes of nuclei (Fig. 2h) changed proportionally with whole-cell volumes; however, the ratio nucleus–cytoplasm remained unchanged in all three lines (Fig. 2k). In summary, the most prominent changes in MDCK cells after up/down-regulation of PV expression consisted in bi-directional changes of mitochondrial volume confirming the inverse regulation of PV and mitochondria in MDCK cells. We assume homeostatic down-regulation of mitochondria resulting from PV overexpression that is reverted by decreasing PV levels in shPV/PV-MDCK cells. In agreement, transcript levels of *Ppargc1a* encoding the mitochondrial master regulator PGC-1α, were ≈ 40% decreased in PV-MDCK cells and after PV-downregulation in shPV/PV-MDCK cells returned to levels similar as in C-MDCK cells (data not shown). Similar results have been previously obtained in WT and PV−/− fast-twitch muscles or in C2C12 myotubes (± PV) [31].

### PV decreases the cell motility due to reduced cell protrusions: lamellipodia, filopodia, microvilli

Mitochondrial Ca^2+^ homeostasis also plays a role in cytoskeleton dynamics and cell migration [66]. Ca^2+^ signals control cell migration by regulating forward movement and cell adhesion [87] and moreover mitochondrial Ca^2+^ uptake controls actin cytoskeleton dynamics and is a prerequisite for efficient cell migration [66]. MDCK cell migration was monitored immediately after seeding (Fig. 3a–c) and individual cells were tracked manually (Fig. 3d, e). Representative movies are shown in Suppl. movies 4–6. The relative velocity (Fig. 3d) of PV-MDCK cells was only half of that of C-MDCK cells (*P* = 0.0190). PV down-regulation in shPV/PV-MDCK cells reverted the relative velocity to values recorded in C-MDCK cells (*P* = 0.8916). Also, the average distance that PV-MDCK cells travelled during the observation period of 2 h was significantly shorter (141.6 ± 10 µm vs. 264.0 ± 22 µm in C-MDCK cells; *P* = 0.0033; Fig. 3e), an effect no longer observed after decreasing PV (shPV/PV-MDCK cells: 278.8 ± 26 µm vs. 264.0 ± 22 µm in C-MDCK; *P* = 0.8698). Additionally the circularity coefficient of cells was estimated 2 h after seeding using phase-contrast images (Fig. 3c, f). PV-MDCK cells were mostly rounded, unlike either C-MDCK (*P* < 0.0001) or shPV/PV-MDCK cells (*P* < 0.0001). From more than 500 cells in 54 fields that were analysed in each group, 16.67 ± 1.33% of cells with round shape, with circularity coefficient ≥ 0.8, were found in control group. While 82.33 ± 3.25% roundish cells were detected in PV-MDCK group (*P* < 0.0001), only 14.00 ± 2.00% round shaped cells were found in the PV/shPV group.

As cytosolic Ca^2+^ pulses are involved in modulating cell directionality [91] and lamellipodia retraction [86], we analyzed protrusions, i.e. filopodia and lamellipodia from TEM images (Fig. 3g, h). PV-overexpressing cells showed almost 50% fewer protrusions compared to C-MDCK and PV/shPV-MDCK cells (*P* < 0.0001). The slightly larger surface area of protrusions in PV/shPV-MDCK compared to C-MDCK cells (*P* = 0.0169) correlated with similar, yet not significant differences in cell mobility between the two cell lines (Fig. 3d, e). Since cell motility and movement are energy-requiring processes, we visualized the mitochondrial network within a small subplasmalemmal region of single live MDCK cells (Fig. 3i). Multiple mitochondria were detected marker-free (without staining) based on differences in the refractive index of different cell components [1, 24]. The relative volume of mitochondria in a given subplasmalemmal compartment of MDCK cells was significantly lower in PV-MDCK cells compared to C-MDCK cells (Fig. 3j; *P* < 0.001). The relative mitochondrial volume in low-PV shPV/PV-MDCK cells was then increased when compared to PV-MDCK cells (*P* < 0.0001) and it was even slightly higher than in C-MDCK cells (*P* < 0.01) indicative of a slight “overshoot”. These data strongly correlate with the ones reporting area of protrusions (Fig. 3h) and relative cell velocity (Fig. 3d).

### PV-overexpression decreases length, surface area and density of mitochondria

**Fig. 4:**
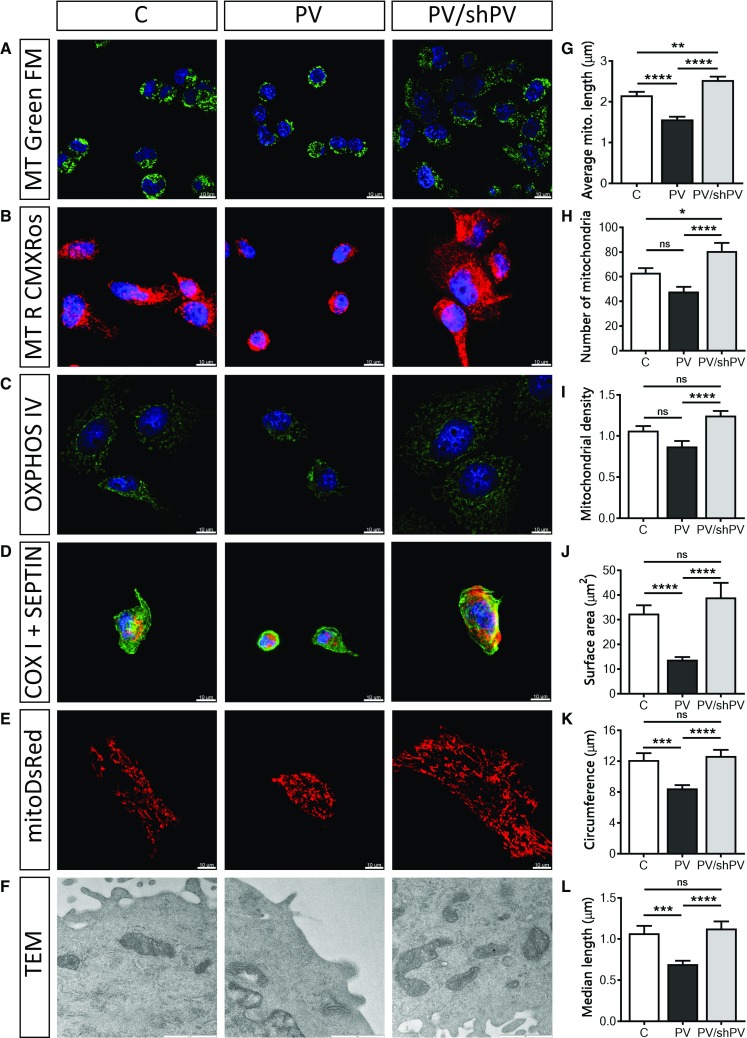
PV-overexpression decreases the length, surface area and density of mitochondria. Several fluorescent dyes, antibodies or fluorescent proteins were used to confirm the mitochondrial morphology and location within cells. Representative images show mitochondria visualized by MitoTracker™ Green (**a**) and MitoTracker™ Red CMXRos (**b**). Immunocytochemical staining using a primary antibody against oxphos IV (**c**) or against COX I (red) and septin 7 (green) (**d**) is shown. Nuclei were counterstained with DAPI (blue). MDCK cells were transfected with the mitochondria-targeted protein mitoDsRed (**e**) to quantify the mitochondrial length (**g**), number (**h**) and density of mitochondria (**i**). More than 1500 mitochondria were analyzed per group. Mitochondrial ultrastructure (**f**) was examined by transmission electron microscopy (TEM) at 33,000× magnification. Scale bar represents 1 µm. The mitochondrial size was determined by measuring the mitochondrial surface area (**j**), circumference (**k**) and median length (**l**) and from at least 30 cells per group with 100 individual mitochondria

Mitochondrial shape and length varies in living cells and ranges from punctate (globular) structures to tubular networks [2]. The overall mitochondrial shape was visualized with the mitochondrial membrane potential-independent dye MitoTracker™ Green FM (Fig. 4a) and the mitochondrial network with an intact membrane potential with MitoTracker™ Red CMXRos (Fig. 4b). Additionally, oxphos IV complex antibodies were used to stain mitochondria (Fig. 4c). To simultaneously track overall cell morphology (green) and mitochondrial structure (red), antibodies against septin 7 and cytochrome c oxidase (COX I) were used, respectively (Fig. 4d). For quantification of the mitochondrial length, the widely used fluorescent mitochondrial marker mitoDsRed was applied (Fig. 4e). Time-lapse confocal images (*xy*-*t* scans) were acquired (frame rate 6/min) to follow mitochondrial movement over time and to facilitate recognition of single mitochondria, thus minimalizing misinterpretation of mitochondrial length. Mitochondrial length (Fig. 4g), the number of distinct mitochondria (Fig. 4h) and mitochondrial density (Fig. 4i) were calculated for each analyzed cell. Mitochondria in PV-MDCK cells were shorter compared to either C-MDCK cells (1.57 ± 0.06 vs. 2.16 ± 0.08 µm, *P* < 0.0001) or shPV/PV-MDCK cells (1.57 ± 0.06 vs. 2.53 ± 0.08 µm, *P* < 0.0001, Fig. 4g). Also, the number of mitochondria per cell counted on the xy-scan images was lower in PV-MDCK cells (48 ± 4 mitochondria in PV-MDCK cell compared to 63 ± 4 mitochondria in C-MDCK cells; *P* = 0.0660, Fig. 4h) and compared to shPV/PV-MDCK cells (48 ± 4 vs. 81 ± 6 mitochondria, *P* < 0001). Mitochondrial density (Fig. 4i) was reduced in the PV group by almost 18% compared to control cells (*P* = 0.0652) and by almost 30% compared to the PV/shPV group (*P* < 0.0001). Ultrastructural analysis demonstrated normal “healthy” mitochondrial morphology (e.g. no swelling) in all MDCK lines (Fig. 4f). In PV-overexpressing MDCK cells, the surface area covered by mitochondria was decreased (13.78 ± 1.08 µm^2^ vs. 32.47 ± 3.36 µm^2^; *P* < 0.0001; Fig. 4j), the circumference of mitochondria was smaller (8.51 ± 1.18 µm vs. 12.03 ± 1.33 µm, *P* = 0.0005; Fig. 4k) and the median length was decreased (0.69 ± 0.04 µm vs. 1.07 ± 0.09 µm; *P* = 0.0004; Fig. 4l) compared to C-MDCK cells. PV-downregulation in shPV/PV-MDCK cells mostly reverted, or even “overpassed” the phenotype to the state observed in C-MDCK cells.

### Parvalbumin decreases mitochondrial velocity and alters mitochondrial dynamics resulting from reduced fusion events

**Fig. 5:**
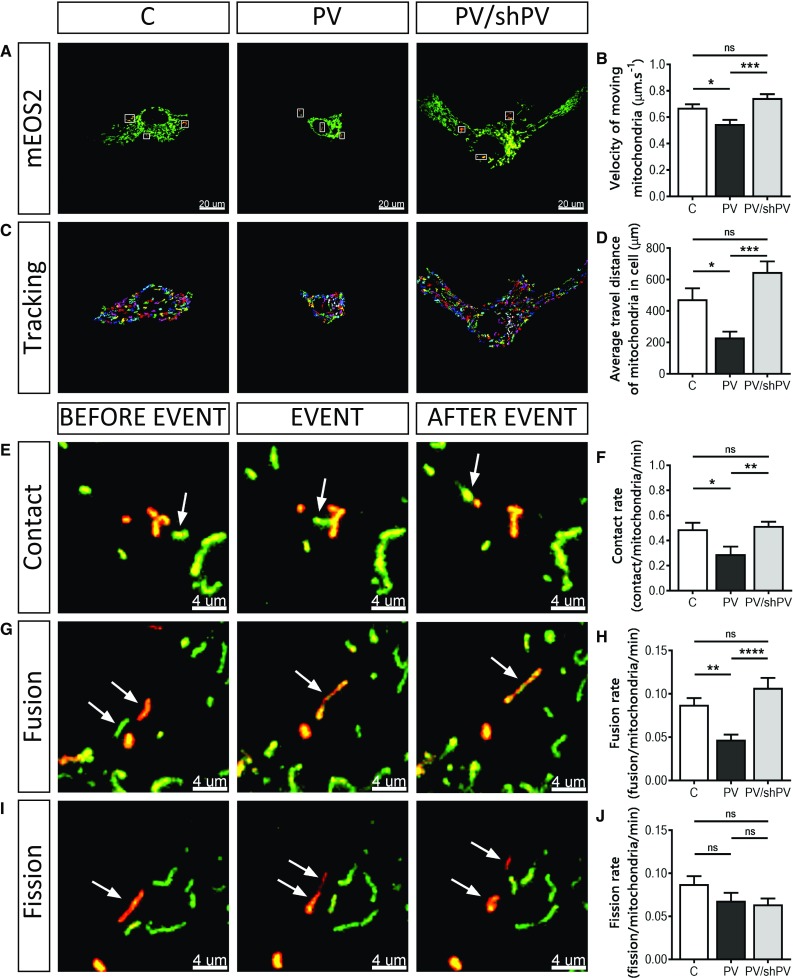
Parvalbumin decreases mitochondrial dynamics. **a** Representative images showing MDCK cells transfected with the mitochondria-targeted, photoconverted fluorescent protein mEOS2. Non-activated mitochondria are shown in green and activated (red) mitochondria are shown within white rectangles. All mitochondria were then tracked in Retrack software version 2.10.05. In this software, tracked mitochondria were marked by colors (one out of the seven colors) to easily follow movement of each mitochondrion. Trajectories were generated from entire *xy*-*t*-stacks shown in the overlap after subtraction of sequential images (**c**). Quantification of mitochondrial velocity (**b**) and measurements of distances that mitochondria travelled within a cell (**d**) are shown. Visualization of mitochondrial contacts (**e**), fusion (**g**) and fission (**i**) events. Mitochondria of interest are marked with white arrows to follow this event in time-lapse mode. Values for mitochondrial contact rates (**f**), fusion rates (**h**) and fission rates (**j**) during 10 min observation were normalized per activated mitochondria per minute

Mitochondria are mobile inside cells and are able to adapt their morphology by fusion and fission events [13, 18]. To track mitochondria and moreover to follow fusion–fission events in living MDCK cells, previously developed methods were applied [9, 10, 20]. All three MDCK cell lines were transfected with a plasmid encoding the photo-convertible fluorescent protein mEOS2 targeted to mitochondria; illumination at 488 nm results in green fluorescent mitochondria. Brief illumination of selected mitochondria (white rectangles) with a 405 nm laser converts the green fluorescence to red (Fig. 5a). Fusion between green- and red-labeled mitochondria resulted in mixed mitochondria characterized by yellow-orange fluorescence (Fig. 5g). On average, nine mitochondria (three separate regions, three mitochondria per region) were photoactivated per cell (Fig. 5a, white rectangles). To avoid photoactivation of mitochondria located in close proximity of the mitochondrion of interest, only mitochondria localized in the cell periphery or clearly separated from mitochondrial clusters were selected for photoconversion, nevertheless selected randomly with respect to size and/or shape. More than 100 individual photoactivated mitochondria pooled from 12 cells were analyzed in each cell line. Images were acquired every 10 s during 10 min and the fate of all mitochondria was followed during the entire time course. The velocity of mitochondrial movement was lower in PV-MDCK cells (0.55 ± 0.03 µm s^−1^ vs. 0.67 ± 0.03 µm s^−1^ in C-MDCK cells, *P* = 0.0185; Fig. 5b). The decreased PV levels in shPV/PV-MDCK cells caused an augmentation of the velocity to values recorded in C-MDCK cells (0.75 ± 0.03 µm s^−1^ vs. 0.67 ± 0.03 µm s^−1^, *P* = 0.1950). Also, the average distance that mitochondria travelled within one cell during 10 min was significantly shorter in PV-MDCK cells (230.8 ± 37.0 µm vs. 474.9 ± 69.0 µm in C-MDCK cells; *P* = 0.0343; Fig. 5c, d). After decreasing PV levels in shPV/PV-MDCK cells, the reversal effect was observed and the distance that mitochondria travelled in shPV/PV-MDCK cells was even longer than in C-MDCK cells, although the difference was not significant (648.4 ± 66.0 µm vs. 474.9 ± 69.0 µm, *P* = 0.1433).

Photoconverted mitochondria were analyzed for mitochondria–mitochondria contacts (contacts between mitochondria not leading to any change in fluorescence of the two mitochondria; Fig. 5e), fusion (orange fluorescence after mixing of mitochondrial matrices; Fig. 5g) and fission events (resulting in two shorter mitochondria when a longer mitochondrion splits apart; Fig. 5i). Quantitative analyses (normalized per activated mitochondria per min) showed a significant reduction of contact rates in PV-MDCK cells (0.29 ± 0.06 contact/mitochondria/min vs. 0.49 ± 0.05 contact/mitochondria/min in C-MDCK cells; *P* = 0.0219, Fig. 5f), which was restored by PV downregulation in shPV/PV-MDCK cells (0.51 ± 0.03 vs. 0.29 ± 0.06 in PV-MDCK, *P* = 0.0086; and 0.51 ± 0.03 vs. 0.49 ± 0.05 in C-MDCK, *P* = 0.9262). PV overexpression also significantly decreased the mitochondrial fusion rate (from 0.09 ± 0.01 fusion/mitochondria/min in C-MDCK cells to 0.05 ± 0.01 fusion/mitochondria/min in PV-MDCK cells, *P* = 0.0066), while in shPV/PV-MDCK cells, fusion rates were very similar to ones observed in C-MDCK cells (0.11 ± 0.01 vs. 0.09 ± 0.01, *P* = 0.2648, Fig. 5h). No significant changes were detected in all three MDCK lines with respect to fission rates (Fig. 5j). Representative movies are shown in Suppl. movies 7–10.

### Parvalbumin affects cell size and organization of mitochondria, but not the cytoskeleton organization

Mitochondria in mammalian cells make use of the actin cytoskeleton for short-range displacements and the microtubule cytoskeleton for longer range movements [62]. Moreover, the 3D cell architecture depends on interactions between actin filaments and the microtubule system [32, 57]. To gain more information about cytoskeletal structures of MDCK cells and possibly observe PV-mediated changes, we co-stained the three MDCK cell lines for actin and tubulin (Suppl. Fig. 2a) or mitochondria and tubulin (Suppl. Fig. 2b). Apart from the differences in cell size, we did not observe striking differences in microtubule assembly or the general organization of the cytoskeleton between different MDCK cell lines (Suppl. Fig. 2a, b), yet microtubules in the smaller PV-MDCK cells were less evenly dispersed throughout the cells as in the C-MDCK cells, but more centered in the perinuclear region.

### Effects on mitochondrial volume and mitochondrial fusion are PV-specific

**Fig. 6:**
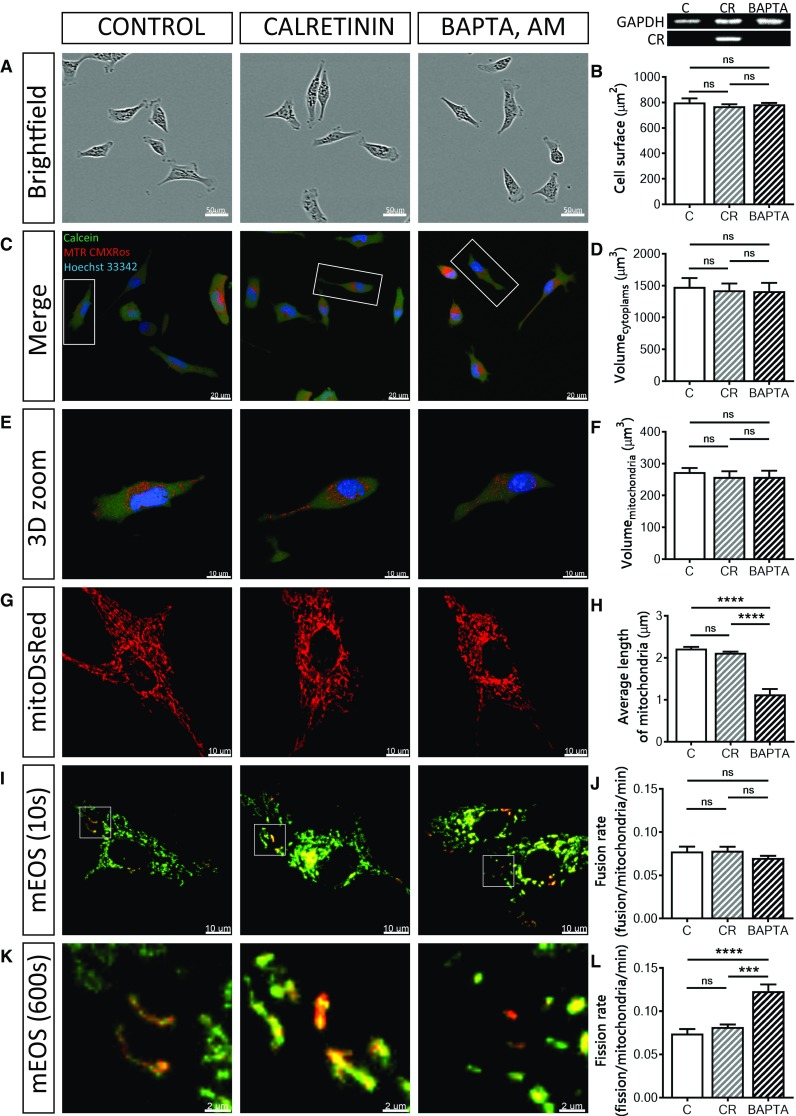
Overexpression of the Ca<Superscript>2+</Superscript> buffer/sensor protein calretinin, and loading of MDCK cells with the non-proteinaceous Ca<Superscript>2+</Superscript> chelator BAPTA. Control MDCK cells were compared to MDCK cells overexpressing calretinin (CR-MDCK) or C-MDCK loaded with 10 µM BAPTA-AM for 30 min (BAPTA). Representative images of MDCK cells acquired using the IncuCyte Live-Cell Imaging system (**a**). Quantification of the surface area of 100 cells in each group (**b**). Protein expression levels of CR in MDCK cell lines were determined also by Western blot analysis. For the normalization, GAPDH signals were used. Representative confocal images (**c**) showing the merged images of the cytoplasm (Calcein-AM), mitochondria (MitoTrackerTM Red CMXRos) and nuclei (Hoechst 33342). Selected representative cells (white rectangles) are shown in 3D view (**e**). Stereological analysis of 3D-reconstructed MDCK cells revealed that the volume of the cytoplasm (**d**) and volume of mitochondria (**f**) was unchanged. Mitochondrial length was measured using the mitochondria-targeted protein mitoDsRed (**g**, **h**). More than 30 cells were analyzed in each group. **i** Representative images showing MDCK cells transfected with the mitochondria-targeted, photoconverted fluorescent protein mEOS2, showing non-activated (green) mitochondria and activated (red) mitochondria. Selected regions (white rectangles) are shown at higher magnification at the end of the experiment (**k**). Estimation of mitochondrial fusion rates (**j**) and mitochondrial fission rates (**l**) were determined during a 10-min observation period and then normalized per activated mitochondria per minute

To address the question, whether the observed effects (changes in cell morphology and mitochondrial volume and dynamics) caused by increased levels of PV were protein-specific, we overexpressed the Ca^2+^-binding protein calretinin (CR), a presumed Ca^2+^ buffer with fast binding kinetics (and additional Ca^2+^ sensor functions [77]) in MDCK cells (CR-MDCK), similarly as in a previous study [6]. Additionally experiments were carried out by loading MDCK cells with the non-proteinaceous fast Ca^2+^ chelator BAPTA-AM (10 µM, 30 min) to investigate the “pure and acute” Ca^2+^ buffering effect on mitochondria. Expression of CR in CR-MDCK cells had no significant effects on cell morphology (Fig. 6a, b) and total cell or mitochondrial volumes (Fig. 6c–f). Moreover, the average mitochondrial length in CR-MDCK cells (2.11 ± 0.03 µm vs. 2.22 ± 0.04 µm in C-MDCK cells; *P* = 0.6603) was not affected (Fig. 6g, h) and also mitochondria fusion and fission rates were unchanged (Fig. 6i–l). A somewhat different picture emerged in BAPTA-loaded and subsequently analyzed MDCK cells. Cell morphology was not altered by short-term BAPTA loading (Fig. 6a–f), however, the average length of mitochondria was significantly decreased to almost half of that of control cells (1.13 ± 0.13 µm vs. 2.22 ± 0.04 µm, *P* < 0.0001, Fig. 6g, h). In line with the shortening of mitochondria caused by BAPTA-AM, the mitochondrial fission rate was increased (0.11 ± 0.01 (BAPTA) vs. 0.08 ± 0.01 fission/mitochondria/min in control cells; *P* < 0.001; Fig. 6k, l), while the fusion rate was unchanged (Fig. 6i, j). The effect of the fast chelator BAPTA on mitochondria is consistent with previous studies [36], where BAPTA-mediated Ca^2+^ buffering was shown to induce mitochondrial cleavage at ER contact sites. In summary, in MDCK cells, none of the PV-mediated effects on cell morphology, mitochondria volume and length, as well as mitochondria dynamics were observed by overexpression of CR, a Ca^2+^ buffer with properties quite distinct from PV [74]. On the other hand, acute Ca^2+^-buffering by BAPTA differently affected mitochondria dynamics when compared to CR: it increased the fission rate leading to shortened mitochondria. Thus, as observed in many instances, (1) alterations brought about by Ca^2+^-binding proteins are protein-specific and (2) effects caused by a particular protein (CR) cannot be completely mimicked by a synthetic non-proteinaceous Ca^2+^ chelator such as BAPTA.

### Parvalbumin reduces mitochondrial mass by increased mitophagy

**Fig. 7:**
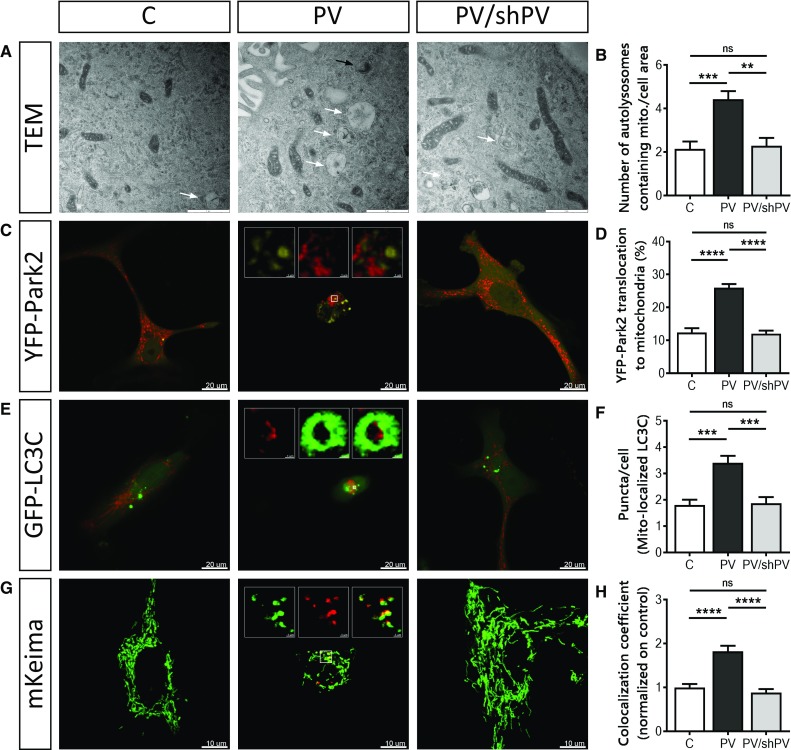
Removal of mitochondria by mitophagy is more pronounced in PV-expressing cells. **a** Representative TEM images depict ultrastructural findings in MDCK cells. Early autophagic vacuoles containing identifiable mitochondria (black arrow) and late autophagosomes containing vesicular structures (white arrows) were observed mostly in PV-MDCK cells. **b** Number of autolysosomes containing mitochondria per cell area. **c** Representative images of MDCK cells transfected with YFP-Park2 (yellow) and mitoKate2 (red) plasmid. When Parkin was translocated to mitochondria, a distinct yellow fluorescence was observed and the quantification is shown (**d**). **e** Representative images of MDCK cells transfected with GFP–LC3C protein (green) together with MitoKate2 (red). Note the brighter green signal staining autophagosomal membranes compared to the weaker cytoplasmic signals. In the white squares a mitochondrion (red) is surrounded by lysosome (green). More green puncta were observed mostly in PV-MDCK cells (**f**). Representative images of MDCK cells transfected with the photoconvertible protein mito-Keima (**g**), allowing identification and quantification (**h**) of normal (green) and acidic (red) mitochondria

The removal of damaged mitochondria by mitophagy is a critical process for maintaining proper cellular functions [52]. TEM images of all three MDCK cell lines revealed early (black arrow) and late (white arrows) mitophagosomes (Fig. 7a). Autolysosomal structures containing damaged mitochondria were clearly more prevalent in PV-MDCK cells than in the two lines with absent (C-MDCK) or lower (shPV/PV-MDCK) PV protein levels (Fig. 7b). One of the key drivers of mitophagy is sustained mitochondrial depolarization below a critical threshold of the mitochondrial membrane potential, which leads to accumulation of PINK1 on the OMM [63] and subsequent recruitment of Parkin to mitochondria [46, 51]. Parkin translocation was investigated in MDCK cells transfected with yellow fluorescent protein (YFP)-tagged Parkin together with the red mitochondrial marker mitoKate2 (Fig. 7c). The percentage of cells showing YFP-Parkin translocation to mitochondria was almost twofold higher in PV-MDCK cells than in either C-MDCK (*P* < 0.001) or shPV/PV-MDCK (*P* < 0.001) cells (Fig. 7d). Specific autophagy receptors such as LC3C located on the mitochondria surface of damaged mitochondria are able to directly interact with membranes of autophagosomes. Since they remain associated with the entire autophagosome, LC3C serves as a key autophagy-related marker for mitophagy [49]. MDCK cells were transfected with plasmids encoding GFP-tagged LC3C (green) and mitoKate2 (red), respectively (Fig. 7e). In most cells, GFP-LC3C expression results in a diffuse green fluorescence signal throughout the cytoplasm and nucleus. In response to macroautophagy-promoting stimuli, the GFP-LC3 signal becomes punctate and predominantly cytoplasmic [7, 52, 60]. The number of GFP-LC3C dots associated with mitoKate2 fluorescence was significantly increased in PV-MDCK cells (Fig. 7e, f; almost twofold increase vs. C-MDCK cells; *P* < 0.001). The distribution of GFP-LC3C puncta in the cytoplasm of PV-overexpressing cells was quite distinct and green dots accumulated mainly in the perinuclear area, while in C-MDCK and shPV/PV-MDCK cells puncta were less numerous and more uniformly distributed throughout the cytoplasm.

To distinguish between healthy mitochondria and ones localized in an acidic environment (lysosomes), the mitochondrially targeted pH-dependent fluorescent protein Keima was used. The excitation spectrum of Keima shifts (from green to red), if mitochondria are trapped in acidic lysosomes [5, 47], as Keima is resistant to lysosomal proteases and thus suitable for measurements of cumulative lysosomal delivery of mitochondria over time [82]. The number of red mitochondria within an acidic environment (Fig. 7g, h) was significantly higher in PV-MDCK cells (*P* < 0.0001) than in MDCK cells with absent-to-low PV levels.

In summary, changes in mitochondrial morphology and the increase of several mitophagy markers in PV-expressing cells indicate a critical involvement of the process of mitophagy linked to the decreased mitochondrial content of PV-MDCK cells.

## Discussion

Both, the Ca^2+^ buffer PV, as well as mitochondria are components of the Ca^2+^-signaling toolkit [4], which in its entirety form the Ca^2+^ homeostasome [76] responsible for the regulation of intracellular Ca^2+^ signals. An intricate interplay between PV and mitochondria has been observed in excitable (neurons, muscle fibers) and non-excitable (epithelial) cells before, entailing a clearly antagonistic mechanism; up-regulation of PV expression leads to a decrease in mitochondrial volume and vice versa by a likely homeostatic mechanism [31, 41]. Ca^2+^ buffering by PV and Ca^2+^ sequestration by mitochondria [68] have rather slow kinetics in comparison to fast buffers such as the presumed Ca^2+^ buffers calbindin D-28k or calretinin [75] and thus still allow for Ca^2+^ signaling in cells with high PV levels or a large mitochondrial volume. Insight on the inverse regulation has been obtained before in (PV-negative) MDCK cells either stably over-expressing PV (PV-MDCK) and in PV/shPV-MDCK cells, where PV overexpression was greatly reduced by an shPvalb approach [41]. Here we set out to gain more mechanistic insight (1) in the PV specificity of the observed changes, (2) on the effects of PV on cell morphology and (3) more importantly on mitochondrial dynamics. With respect to the specificity of the changes mediated by PV: all of the effects caused by increased PV levels in PV-MDCK cells are linked to the particular slow-onset Ca^2+^-buffering properties of PV, since none of the effects were observed in MDCK cells overexpressing the homologous EF-hand protein calretinin with fast Ca^2+^-buffering kinetics [35]. How a given Ca^2+^ buffer (better termed Ca^2+^ signaling modulator; PV, CR) affects intracellular Ca^2+^ signals (e.g. as shown for Ca^2+^ signals in Purkinje cells without PV or calbindin D-28k in the respective knockout mice) [73], then likely translates into differences in the activation of downstream pathways (calmodulin—CaM, and CaM-dependent kinases—CaMKs), finally leading to the observed cellular responses. The fact that also cell morphology was unchanged in CR-MDCK cells (as opposed to PV-MDCK cells) indicates that pathways controlling mitochondria volume and overall cell morphology are coupled. Of note, although Ca^2+^-buffering properties of CR and the synthetic chelator BAPTA are described as rather similar (see Table 2 in [74]), their effects in MDCK cells demonstrate noticeable differences. While overexpression of CR had no effect on mitochondria fission/fusion, BAPTA increased the fission rate resulting in shorter mitochondria.

The down-regulation of PV in shPV/PV-MDCK cells led to a cellular phenotype closely resembling, but not being identical to the one of C-MDCK cells. Thus, the described processes leading to changes in cell morphology and mitochondria structure and dynamics are reversible and highly dynamic, suggestive of a homeostatic mechanism. In most cases, the differences between C-MDCK cells and shPV/PV-MDCK cells might be described as an “overshooting” effect; shPV/PV-MDCK cells were slightly larger (surface, volume, nuclei), contained more, larger (volume) and longer mitochondria, in particular subplasmalemmal mitochondria than the parental C-MDCK cells. Of note, parameters linked to mitochondria dynamics were not significantly different between the two groups. Although PV levels in shPV/PV-MDCK cells (≈ 30 µM) were almost tenfold lower than in the PV-MDCK cells, they are still in a “physiological” concentration range as present, e.g. in hippocampal basket cells (≈ 12 µM; [33]). Thus, it cannot be excluded that lower levels of PV result in opposite morphological changes (larger flatter cells with more mitochondria) than the effects mediated by higher PV levels (smaller, rounder cells with decreased mitochondrial volume) as seen in PV-MDCK cells. Such a hypothesis might be tested in PV-expressing neurons in wildtype and PV−/− mice. While in high PV-expressing Purkinje cells, the absence of PV in PV−/− mice results in a 40% increase in mitochondrial volume in the soma [17], a similar study has not been carried out in the much smaller interneurons, e.g. in cerebellar or hippocampal basket cells. It will be interesting to see whether the absence of PV entails the same (or opposing) effects in “low-PV” hippocampal basket cells than in “high-PV” cerebellar basket cells.

Mitochondrial volume homeostasis is a housekeeping function also essential for maintaining the structural integrity of these organelles. The mitochondrial volume is controlled by the osmotic balance between the cytosol and mitochondria and moreover Ca^2+^ ions play an important role in the regulation of mitochondrial volume [3, 25, 55]. Besides the previously described changes in mitochondrial volume brought about by modifications in PV expression levels, additional unexpected morphological changes were observed in MDCK cells ectopically expressing PV. Besides the obviously visible rounding of the cells, whole cell volumes in PV-MDCK cells (− 30%) were decreased approximately proportional to the decrease in mitochondrial mass (− 40%). This effect might be linked to the mitochondrial Ca^2+^ buffering capacity, shown to be implicated in cytoskeleton dynamics [66] and also to the intracellular distribution of mitochondria, which strongly depends on mitochondria–cytoskeleton interactions [45, 50]. In line with our findings, blocking of mitochondrial Ca^2+^ uptake in MCU-knockdown cells results in an increased circularity coefficient [66]. Accordingly, we had reported before that in the PV-MDCK cells *Mcu* transcript levels are decreased [41]. Of note, the decrease in cell volume cannot be accounted for entirely by the reduction in mitochondrial volume, but is also the result of a decreased volume of cell nuclei. This is supported by the unchanged ratio of *V*_nucleus_/*V*_cytoplasm_. Currently, we do not have a satisfactory explanation by which means the volume of a cell’s nucleus is affected by PV overexpression; yet a similar effect (although not significant) is seen in PV-overexpressing C2C12 muscle cells [31]. While it is obvious that the rather flattened morphology of either C-MDCK cells or shPV/PV-MDCK cells results in a higher surface area than the roundish PV-MDCK cells, also the relative surface area of protrusions (filopodia, lamellipodia) was clearly higher in cells with absent-to-low PV expression. This is likely associated with the increased mobility of these cells. In support, silencing of the *MCU* gene in human breast cancer and HeLa cells leads to an increase in actin cytoskeleton stiffness, loss of cell polarity, as well as impairment of focal adhesion dynamics [66].

Although mitochondria function is assumed to be very similar in all cells, mitochondrial morphology and intracellular distribution are highly variable between tissues and cell types [2, 23, 37, 61]. The number and size of mitochondria in a given cell is the result of several processes: mitochondrial biogenesis, fusion and fission and mitophagy [9, 27, 28]. Analysis of mitochondrial morphology revealed several changes in PV-MDCK cells. In general, mitochondria were shorter, had a smaller surface area and a decreased circumference. In contrary to expectations, the velocity of the moving (smaller) mitochondria in PV-MDCK cells was lower than in the other two lines with none or low PV expression levels. Consequently, also the average travel distance and the contact rates between mitochondria were lower in PV-MDCK cells. Considering that mitochondrial velocity and mitochondrial contacts are important parameters determining mitochondrial fusion, it was not surprising that mitochondrial fusion rates were decreased. A link between mitochondrial velocity and fusion had been observed in several previous studies [10, 15, 29, 56], also highlighting the relevance of Ca^2+^ dynamics in the modulation of mitochondrial mobility [9, 29]. With respect to fission, the rates were unaffected by the presence or absence of PV.

Our study also revealed additional mechanistic details on the effect of PV in decreasing mitochondrial volume. While higher levels of the mitochondrial master regulatory gene PGC-1α, result in a higher fractional volume of mitochondria [34, 88, 90], increased PV levels in PV-MDCK cells were found to result in lower PGC-1α (*Ppargc1a*) transcript levels suggesting that PV-mediated changes in Ca^2+^ signals might also regulate PGC-1α. The regulation in the other direction has previously been observed in PV-expressing fast-twitch muscle *tibialis anterior* (TA) of transgenic mice overexpressing PGC-1α. While mitochondrial volume is increased, expression levels of PV are decreased [34]. This indicates an inverse, antagonistic regulation also of PV and PGC-1α with a likely contribution of Ca^2+^ signal transcriptional modulators such as Ca^2+^/calmodulin kinase kinase α (affecting sirtuin 1 expression, e.g. increased in TA of PV−/− mice) or Ca^2+^/calmodulin kinase II (CaMKII); the latter also up-regulated in TA of PV−/− mice [31].

In PV-MDCK cells, we observed increased YFP-Parkin2 translocation to mitochondria accompanied with increased mitophagy. As reported before, Parkin recruitment to mitochondria [46, 51] as the result of PINK1 accumulation on the surface of depolarized mitochondria [63] is in good agreement with results from our previous study reporting that the collapse of the mitochondrial membrane potential by CCCP occurred at lower concentrations in PV-MDCK cells [41], indicative of a more depolarized state in PV-MDCK mitochondria. Also, the higher amount of GFP-LC3C puncta in PV-overexpressing cells is in support of increased mitophagy. LC3 remains associated with the completed autophagosome and is often considered as the key autophagy-related marker [52, 60]. Moreover, the number of autolysosomes containing mitochondria was increased in PV-MDCK cells indicating increased mitophagy.

We cannot completely exclude the possibility that PV overexpression induced a generic cellular stress response, as it was shown before that CR and calbindin-D28k, but not PV protect against glutamate-induced delayed excitotoxicity. Such excitotoxic effects leading to neuronal cell degeneration are often accompanied by a prolonged increase in the intracellular level of Ca^2+^ ions and l-glutamate-induced toxicity is assumed to be mediated via a Ca^2+^-dependent mechanism [26, 59]. Increased Ca^2+^ buffering by PV appears neuroprotective under conditions of short-term excitotoxicity [89], in vivo; after prolonged periods, two other mechanisms, increased Ca^2+^ shuttling by PV and downregulation of mitochondrial volume, actually aggravate the excitotoxic effects [59].

In conclusion, PV expression strongly affects overall morphology and mobility of MDCK cells. We conjecture that most of these effects are mediated through modulation of mitochondrial volume and dynamics. Alterations in mitochondrial dynamics and mitochondria turnover in PV-MDCK cells is associated with mitochondrial shortening, reduced fusion and augmented removal of damaged mitochondria by mitophagy. While the overall decreased mitochondrial mass contributes to the smaller cell size of PV-MDCK, the shrinking of the volume of the nuclei by yet unknown mechanisms is an additional unexpected observation. Finally, all effects are PV-specific, since none of the described changes are seen in MDCK cells expressing the homologous fast Ca^2+^ buffer CR. Our results provide a novel insight into the complex crosstalk between PV and mitochondria regulation through changes in mitochondrial dynamics.

## Materials and methods

### Cell culture

Madin–Darby canine kidney (MDCK) cells were cultured in Dulbecco’s modified Eagle’s medium with high glucose (Gibco, Switzerland) and supplemented with 10% heat-inactivated fetal calf serum (Gibco, Switzerland) and 100 U/ml Penicillin and 100 µg/ml Streptomycin, as described previously [41]. Three MDCK cell lines were used in our study as reported before [41]: (1) control MDCK cells (C-MDCK), (2) MDCK cells with stable ectopic expression of parvalbumin (PV-MCDK) mediated by the lentiviral vector (pLVTHM-PV, #12247, Addgene), (3) PV-MDCK cells, where PV expression was constitutively downregulated by a short-hairpin RNA 100% identical to the sequence of dog PV (pLKO.1-Pvalb) resulting in the cell line shPV/PV-MDCK. Calretinin expression was achieved using the lentiviral system pLVTHM (Addgene plasmid #12247) as described previously [6]. All cell lines were regularly checked to avoid mycoplasma contamination.

### Confocal microscopy: 3D measurement

MDCK cells were seeded on collagen-coated glass bottom dishes (MatTek Corp., Ashland, MA) at a density of 1 × 10^4^ cells/cm^2^ and allowed to grow for 2 days. At the day of experiments, MDCK cells were loaded with 1 µM Calcein-AM (C3100MP, Molecular Probes), 500 nM MitoTracker™ Red CMXRos (M7512, Molecular Probes) in DPBS (Sigma) for 30 min and with 1 µM Hoechst 33342 (H1399, Molecular Probes) for the last 5 min before live cell imaging. A laser scanning confocal microscope Leica TCS SP5 equipped with motorized conventional Galvo stage was used in this study. Optical sections were acquired along the *Z*-axis at 0.42 μm step size using a 40 × oil-immersion APO Plan objective with 1.3 numerical aperture. The parameters of acquisition were as follow; image format of 1024 × 1024 pixels, 200 Hz scan speed and pinhole diameter was set to 1 AU. Voxel size was 0.379 × 0.379 × 0.420 µm. Measurements were performed at room temperature in DPBS without Ca^2+^ and Mg^2+^ to reduce mitochondrial movement [19]. Calcein-AM was excited using a 488-nm argon laser, MitoTracker™ Red CMXRos using a 561-nm DPSS laser and Hoechst33342 using a 405-nm laser. Fluorescence emission was recorded at 419–474 nm (Hoechst 33342), 510–554 nm (Calcein-AM) and 584-683 nm (MitoTracker™ Red CMXRos) in a sequential mode with lowest possible laser intensity to minimize photobleaching. In each daily experiment, three dishes per cell line were scanned. In each dish, images from three randomly selected regions were acquired. All experiments were performed three times. More than 400 cells in each group were measured.

### 3D reconstruction and morphometric analysis

For image reconstruction and volumetric analysis, complete series of z-stack images were processed using the commercially available software Imaris 9.0.1^®^ (Bitplane, AG). To measure volumes of whole cells, nuclei or mitochondria “ImarisCell” and “ImarisSurface” software packages were used. Identical parameters and algorithm settings were applied for each cell in the three investigated groups. Only well visible, single nuclei cells were chosen for further analysis. Mitotic cells or cells located at the edges of the visual field were excluded from analysis. Statistical analyses were performed directly in Imaris software using “Imaris Measurement Pro” and “Imaris XT” licenses.

### Live cell imaging, cell tracking and estimation of circularity coefficient

MDCK cells were analyzed using the IncuCyte live-content imaging system (Essen Bioscience). Briefly, MDCK cells were seeded at a density of 1 × 10^2^ cells/cm^2^ in 96-well plates (Essen Bioscience) and images were automatically acquired immediately after seeding with an image acquisition rate of 12/h for 2 h at 20× magnification. MDCK cells were tracked manually using the open source TrackMate plugin of Fiji software, which allows to quantify movement of objects between frames of a 2D-stack and retrieve *XY* coordinates together with velocity or distance covered between two frames [6, 85]. The circularity coefficient of cells was estimated using the phase-contrast images obtained in the same experiments. Quantification of the circularity coefficient was carried out similarly as described in [66]. Ranges from 0 to 1 (0 indicates elongated polygon; 1 indicates perfect circle) were obtained using the ImageJ software, where the circularity coefficient was analyzed as 4*π* (area)/perimeter^2^.

### Tomography

For non-invasive marker-free imaging of live cells, in particular imaging of mitochondrial networks a 3D-holographic and tomographic microscope 3D Cell Explorer (Nanolive, Switzerland) was used as reported before [1, 24]. MDCK cells were seeded on collagen-coated glass bottom dishes (MatTek Corp., Ashland, MA) at a density of 1 × 10^4^ cells/cm^2^ and visualized using a 60× dry objective (NA 0.8) with a class 1 low-power laser (*λ* = 520 nm, sample exposure 0.2 mW/mm^2^) with a 3D-tomography frame rate of 0.5 frames/s with full self-adjustment and ∆*z* = 400 nm. Reconstruction and volumetric analysis, complete series of z-stack images were carried out using the commercially available STEVE software (Nanolive, Switzerland).

### Fluorescence staining and immunocytochemistry

For live-cell imaging, MDCK cells were loaded with 100 nM MitoTracker™ Green FM (Thermo Fisher, Scientific) in DPBS for 30 min at 37 °C. To stain the nuclei, Hoechst^®^ 33342 (Thermo Fisher, Scientific) was added for the last 5 min. For immunocytochemistry, mitochondria in fixed cells were stained using MitoTracker™ Red CMXRos (500 nM) according to manufacturer’s specifications. Nuclei were counterstained with DAPI. MDCK cells of all three lines were seeded onto sterile glass coverslips in 12-well culture plates at a density 1 × 10^4^ cells/well and incubated with the appropriate medium until 70–80% confluence was reached. Cells were then washed twice with pre-warmed 0.1 M TBS (Tris-buffered saline), fixed with 4% paraformaldehyde solution for 10 min at 37 °C and incubated in blocking buffer (0.1 M TBS containing 10% donkey serum and 0.4% Triton-X100) for 60 min at RT. Cells were incubated overnight at 4 °C with the primary antibodies. The following antibodies were used at the indicated dilutions: rabbit polyclonal anti-OXPHOS IV (Alomone Labs, 1:250), mouse monoclonal anti-cytochrome oxidase I (COX I Molecular Probes, Invitrogen AG, 1:1000), rabbit polyclonal anti-Septin7 (Bethyl Laboratories, LubioScience, 1:5000), guinea pig anti-PV690 (Swant, Marly, Switzerland 1:1000), mouse monoclonal anti-actin (Santa Cruz, C-2 sc-8432, 1:50), rabbit monoclonal anti α-tubulin (Cell Signaling technology, #2125, 11H10, 1:50). After washing, MDCK cells were further incubated with Alexa Fluor 488 (Jackson Immunoresearch Laboratories, 1:400), Alexa Fluor 594 (Jackson Immunoresearch Laboratories, 1:400), or Alexa Fluor 647 (Jackson Immunoresearch Laboratories, 1:400) conjugated secondary antibodies for 3 h at RT. At last, cell nuclei were counterstained with DAPI (Molecular Probes, 5 µg/ml) for 5 min, then mounted with Hydromount solution (National Diagnostic, Atlanta, GA) and examined with a Leica TCS SP5 confocal microscope.

### Transfection

MDCK cells growing on 35-mm glass-bottom dishes were co-transfected the day after plating using transient transfection methodology by LipofectamineTM 2000 (Invitrogen). Conditioned medium was removed from dishes and 120 µl of Opti-MEM^®^ I medium containing 1.5% Lipofectamine™ 2000 with the appropriate concentration of plasmid DNA was added to the glass ring and incubated for 3 h, thereafter 2 ml of complete media was added to the dishes. The following plasmids were used at the indicated concentrations: mitoDsRed (0.25 µg), mEOS2 (0.3 µg), mitoKate2 (0.1 µg), YFP-Park2 (0.3 µg), GFP-LC3-C (0.25 µg), mitoKeima (0.3 µg). MDCK cells were then cultured for 2 days with mitoDsRed, mEOS2, mitoKate2, YFP-Park2, GFP-LC3-C or 4 days with mitoKeima to enable expression of the transfected DNA. Cells were kept in DPBS (no Ca^2+^, Mg^2+^) at RT when mitochondrial movement was not required or in DPBS containing Ca^2+^ and Mg^2+^ at 37 °C to allow for mitochondrial movement.

### Morphometric analysis of mitochondrial length

For whole-cell mitochondrial length experiments, MDCK cells were transfected with mito-DsRed and examined 2 days after transfection using a confocal microscope Zeiss LSM 780 (Carl Zeiss Microscopy GmbH, Göttingen, Germany). MDCK cells were selected randomly; one cell was acquired at a time using a 63× water immersion objective (LCI Plan-Neofluar, 1.3 NA, immersion-corrected DIC M27). During the whole experiment, MDCK cells were maintained in complete media under physiological conditions in controlled atmosphere, temperature and humidity (e.g. 37 °C, 5% CO_2_) using the Carl Zeiss large incubation system to allow for mitochondrial movement. To follow and recognize a single mitochondrion, a time-lapse series of confocal images were recorded. Images were acquired every 10 s as reported previously [20]. Mitochondria labeled with mito-Kate2 were visualized using a 561 nm DPSS laser. Morphometric analyses were performed using ZEN imaging software (Carl Zeiss Microscopy GmbH, ZEN—black version), length of mitochondria and number of mitochondria were extracted, and then averaged per cell. The ratio of total length of mitochondria and cell area was calculated. The latter represents a proxy measure of mitochondria density. Mitochondria from at least 27 cells per line were measured (three fields per dish, three dishes per group in one experiment, experiments performed in triplicates).

Mitochondrial ultrastructure was assessed by transmission electron microscopy (see below). Blind analysis of randomly numbered electron micrographs were analyzed by blinded experimenters using the ImageJ software. For each mitochondrion, median length, circumference of the outer mitochondrial membrane and surface area was measured using the Wand Tool in ImageJ without knowing the identity of the analyzed MDCK line. Only after completion of all the counting procedure the attribution of a given image to one of the three cell lines was revealed.

### Transmission electron microscopy (TEM)

MDCK cells were prepared for TEM analysis as described previously [42, 83]. MDCK cells were seeded on PET track-etched membranes with 3-μm pores (Becton–Dickinson AG, Allschwil, Switzerland) at a density of 3 × 10^5^ cells/cm^2^ and grown to a semi-confluent state in normal media. Cells were fixed with pre-warmed fixation buffer containing 2.5% glutaraldehyde in 0.1 M Na-cacodylate-HCl (pH 7.4) at RT for 30 min and rinsed with 0.1 M Na-cacodylate-HCl buffer (pH 7.4). After post-fixation in 1% OsO_4_ for 20 min, cells were embedded in Epoxy resin. The dehydration procedure was carried out through gradated ethanol concentrations (50–100%). Finally, membranes were cut as ultrathin sections (70 nm) and mounted on aluminum sample holders. For visualization, a Philipps CM100 Biotwin scanning electron microscope equipped with MORADA camera was used. Micrographs were taken with the iTEM software (Olympus Soft Imaging Solutions GmbH). The experimenter captured images randomly, without knowing the experimental group. Images were taken at a final magnification of 33,000× (mitochondria) or 24,500× (autophagosomes). Resulting *X*, *Y* calibration was 1.188 nm/P for a magnification of 33,000× or 1.157 nm/P for a magnification of 24,500×, respectively. Image resolution was 1854 × 1336 pixel.

### Mitochondrial fusion–fission

MDCK cells were transfected with a bright and photostable photoconvertible fluorescent protein mEOS2. MDCK cells were seeded at a density of 1 × 10^4^ cells/cm^2^ on glass bottom dishes and transfected the next day. MDCK cells were incubated for 48 h to allow for the accumulation of mEOS2 expression. Mitochondria were visualized using a laser scanning confocal microscope Zeiss LSM 780, with a 63×/1.3 water immersion objective. To avoid unwanted shift of focus, automated perfect focus stabilization was permitted. Temperature and atmosphere during experiments were controlled using large incubation systems (Zeiss). Mitochondria-targeted mEOS2 was illuminated with a 488-nm argon laser line to visualize green mitochondrial fluorescence. Randomly selected peripheral mitochondria (2–3 mitochondria per ROI, three ROIs per cell) were photoconverted to red using a 405-nm diode laser and illuminated using a 561-nm DPSS laser. Images were taken at 10-s intervals for 10 min. Events of all activated mitochondria (more than 100 mitochondria per line) were followed throughout the time-lapse movie, and the fusion and fission events were recorded. Three different cells were visualized per dish and experiments were performed five times. Thus, the analyzed mitochondria populations originate from 15 different cells for each line. Mitochondria were further tracked manually using freeware software Retrack version 2.10.05, as described earlier [20, 54]. Retrack software marks automatically each mitochondrion with one out of seven different colors, to easily follow individual mitochondria and to distinguish them from mitochondria that had been already tracked.

### Mitophagy assay

Mitophagy was assessed by different approaches. On TEM images early or late mitophagosomes were searched and quantified. Mitophagosomes and autolysosomal structures were identified from TEM images according to criteria described before [39, 40, 44]. Several marker proteins involved in mitophagy were identified by confocal microscopy. First Parkin translocation to mitochondria was investigated. MDCK cells were transfected with an YFP-PARK2 plasmid [20, 21] together with the mitochondrial marker mitoKate2. Images were randomly captured using a ZEISS LSM 780 confocal microscope. Colocalization of YFP-PARK2 aggregates with mitoKate2 was analyzed using the “Imaris Colocalization” module. At least 30 cells from three independent experiments were analyzed per line.

To visualize autophagosomes, cells were transfected with the GFP-LC3C plasmid. Cells transfected with a GFP-LC3-encoding construct usually exhibit a diffuse green fluorescence covering the entire cytoplasm. When autophagy occurs, the GFP-LC3 signal changes to a punctate pattern. Thus, the increase in the number of GFP-LC3 punctae is an indicator of autophagosomes in cells. Quantification of GFP-LC3C punctate patterns co-localized with mitoKate2 was carried out as described before [9, 21].

Finally, we used the mitochondria-targeted fluorescent protein Keima, known to be able to shift its excitation spectrum from 440 nm (mitochondria at neutral pH) to 586 nm, if mitochondria are delivered to acidic lysosomes [5, 47]. Images were acquired using a laser scanning confocal microscope using the laser lines 458 nm for mitochondria at neutral pH, and 561 nm for mitochondria exposed to an acidic pH. In each experiment, ten images were randomly captured per dish and experiments were repeated three times. Quantification was carried out as reported previously [9, 20]. The mitophagy level was then estimated by quantification of the total number of red pixels divided by the total number of all pixels as suggested previously [82].

### Western blot analyses and estimation of intracellular PV concentrations

MDCK cells were collected and homogenized in ice-cold PBS, prior to freezing at − 20 °C. Pellets were resuspended in RIPA-buffer with Complete Protease Inhibitor Cocktail (04693116001, Roche) and centrifuged at 30,000*g* at 4 °C for 15 min. The protein content was measured using the DC™ protein assay kit (500-0111, BioRad) and equal amounts of protein were denatured in Pierce Lane Marker Reducing sample buffer (#39,000, Thermo Fisher) for 5 min at 99 °C, resolved on 4–20% Mini-PROTEAN^®^ TGX™ precast gel (456-1094, Biorad) and transferred onto Immobilon^®^P^SQ^ membrane (ISEQ00010, Millipore) in 0.1 M Tris-base, 0.192 M glycine, and 20% (v/v) methanol using an electrophoretic transfer system. Membranes were blocked in Odyssey blocking buffer (LICOR Bioscience, 927-40000) at RT for 1 h. After blocking, the membranes were incubated sequentially overnight with different primary antibodies based on the requirement of the experiment. Antibody dilutions used were as followed: guinea pig anti-PV690 1:10,000 (Swant, Marly), rabbit anti-GAPDH 1:10,000 (Sigma G9545), rabbit anti-CR7696 1:10,000 (Swant, Marly). Then membranes were incubated with the appropriate secondary antibody. For PV and GAPDH detection, horseradish peroxidase-coupled secondary antibodies (Sigma) were used at a dilution of 1:10,000 for 1 h at RT. The protein bands were visualized with the Immobilon Western AP Substrate (Millipore, Zug, Switzerland). Imaging and analysis of the blots were performed with the FluorChem E system (Cell Biosciences, Santa Clara, USA). Otherwise, goat anti-mouse IRDye 800 CW (926-32210) or goat anti-rabbit IRDye 680 LT (926-68021, all from Li-Cor Bioscience) for 1 h at RT. Immunoreactive bands and molecular weight markers were detected using the Odyssey Infrared Imaging System (Li-Cor Bioscience, Lincoln, NE, USA). For determining the PV concentration in the different MDCK cell lines, parallel cultures were analyzed. In one the total amount of protein was determined and in the other one the number of cells (in the order of 10^6^ cells/flask) was counted resulting in the amount of protein per cell. Using a calibration curve of pure PV (Suppl. Fig. S1), we calculated the amount of PV per cell and after determining the average cell volume (Fig. 2j), the intracellular PV concentration was calculated.

### Statistical data analysis

All data are shown as mean ± standard error of mean (S.E.M), from at least three independent experiments. GraphPad Prism 6 software was used for statistical comparisons and generation of the graphs. The D’Agostino–Pearson omnibus test was used as normality test. One-way ANOVA followed by the Bonferroni posttest were used for normally distributed data, otherwise Mann–Whitney *U* test or Kruskal–Wallis tests followed by Dunn’s test were used to compare differences between experimental groups. The *P* values were assigned as *P* < 0.05*, < 0.01**, < 0.001***, *P* < 0.0001****.

## Electronic supplementary material

Below is the link to the electronic supplementary material.

Supplementary movies 1–3. Visualization of representative MDCK cells in 3D. **1,** control MDCK cell; **2,** MDCK cell with PV-overexpression; **3,** MDCK cell, in which ectopic expression of PV was downregulated by shRNA. In each movie the cytoplasm is shown in green and nuclei in blue. Mitochondria stained by MitoTracker™ Red CMXRos were reconstructed using the isosurface mode in Imaris and are shown in red. Note the differences between mitochondrial size, structure and position, as well as cell size and attachment to the surface of the dishes (AVI 7007 kb)

Supplementary material 2 (AVI 5995 kb)

Supplementary material 3 (AVI 7620 kb)

Supplementary movies 4–6. Representative time-lapse images showing motility of MDCK cells **4,** C-MDCK cell; **5,** PV-MDCK cell; **6,** shPV/PV-MDCK cell. Cells were seeded and in IncuCyte images were taken every 5 min during 2 h. For movies JPG compression was used. Note the differences between cell size, structure and position of cells at the beginning of the experiment (0 min) and at the end (120 min) (AVI 508 kb)

Supplementary material 5 (AVI 474 kb)

Supplementary material 6 (AVI 467 kb)

Supplementary movies 7–10. Time-lapse movies of non-activated (green) and activated (red) mitochondria visualize mitochondrial movement and dynamics in representative C-MDCK (**7**), PV-MDCK (**8**), shPV/PV-MDCK (**9**) cells as well as in selected regions shown at higher magnification (**10**). Images were acquired every 10 s during 10 min (AVI 3284 kb)

Supplementary material 8 (AVI 2305 kb)

Supplementary material 9 (AVI 4279 kb)

Supplementary material 10 (AVI 1358 kb)

Supplementary Fig. S1 Estimation of the PV concentration in MDCK cells. **a**) Detection of protein expression levels for PV (M_r_:12 kDa) and GAPDH (M_r_:35 kDa) in C-MDCK cells, PV-MDCK cells and PV/shPV-MDCK cells by Western blot analysis. Increasing amounts of purified PV (2, 5, 10, 15, 20, 25 ng) were used for PV determination in MDCK cells. **b**) Analysis of PV Western blot signals in MDCK cells. PV expression was below the threshold for detection in C-MDCK cells. A clear signal for PV was visible in PV-MDCK cells, as shown from a representative Western blot (**a**). PV expression of PV-MDCK cells was set as 100%, thus PV/shPV-cells expressed 10.43 ± 0.88% of PV protein compared to PV-MDCK cells. Determination of the quantity of PV per MDCK cell was estimated from the calibration curve showing increasing amounts of pure PV (**c**). According to the calibration curve, PV protein amounts in PV-overexpressing MDCK cells is equal to 5.77 ± 0.88 ng per cell and to 0.78 ± 0.38 ng in PV/shPV-MDCK cells (**d**) (PDF 400 kb)

Supplementary Fig. S2 Subcellular localization of actin and tubulin in MDCK cells. MDCK cells were plated for 24 h, fixed and stained for α-actin, α-tubulin and DAPI. **a**) Representative images show single Z-sections at the height of the largest diameter of the nucleus (DAPI, blue), actin (green) and tubulin (red) in fixed MDCK cells. **b**) MDCK cells were plated for 24 h, then loaded with MitoTrackerRed CMXRos, washed three times and then fixed and stained for α-tubulin and DAPI. Representative images of the nucleus (DAPI, blue), mitochondria (magenta) and tubulin (green) showed the organization of microtubules together with the distribution of mitochondria on microtubule tracks (PDF 9820 kb)

## Abbreviations

Ca^2+^: Calcium ions
CaMKII: Ca^2+^/calmodulin kinase II
CCCP: Carbonyl cyanide *m*-chlorophenyl hydrazine, mitochondrial oxidative phosphorylation uncoupler
C-MDCK: Control MDCK cells
COX I: Cytochrome c oxidase I
GAPDH: Glyceraldehyde-3-phosphate dehydrogenase
CaM: Calmodulin
CaMK: Calmodulin-dependent kinase
GFP: Green fluorescent protein
Dnm1: Dynamin 1
Drp1: Dynamin-related protein 1
Efhd1: Mitocalcin, EF-hand domain family member D1
IMM: Inner mitochondrial membrane
Mcu: Mitochondrial calcium uniporter
Mcur1: Mitochondrial calcium uniporter regulator1
MDCK cells: Madin–Darby canine kidney cells
mEOS2: Mitochondrially targeted photostable photoconvertible fluorescent protein
Mfn1: Mitofusin 1
Mfn2: Mitofusin 2
Mg^2+^: Magnesium ions
Micu1: Mitochondrial calcium uptake 1
mitoDsRed: Mitochondria-targeted red fluorescent protein
mKeima: Mitochondria-targeted Keima fluorescent protein
OMM: Outer mitochondrial membrane
OPA1: Dynamin-related GTPase OPA1
PARK2: Ubiquitin ligase Parkin
PGC-1α: Peroxisome proliferator-activated receptor γ coactivator-1 alpha
PINK1: Mitochondrial kinase PTEN-induced putative kinase 1
PV: Parvalbumin
PV-MDCK: Cells with ectopic PV expression
shPV: Constitutive down-regulation of PV by shRNA
SIRT1: Sirtuin 1
TA: Tibialis anterior
TEM: Transmission electron microscopy
Ucp2: Uncoupling protein 2
YFP-Park2: Yellow fluorescent protein Parkin
