# Supplementary figures and images for: Parvalbumin alters mitochondrial dynamics and affects cell morphology
Source: Cell Mol Life Sci. 2018 Sep 25;75(24):4643–66. doi: 10.1007/s00018-018-2921-x (PMC6208788; doi:10.1007/s00018-018-2921-x)

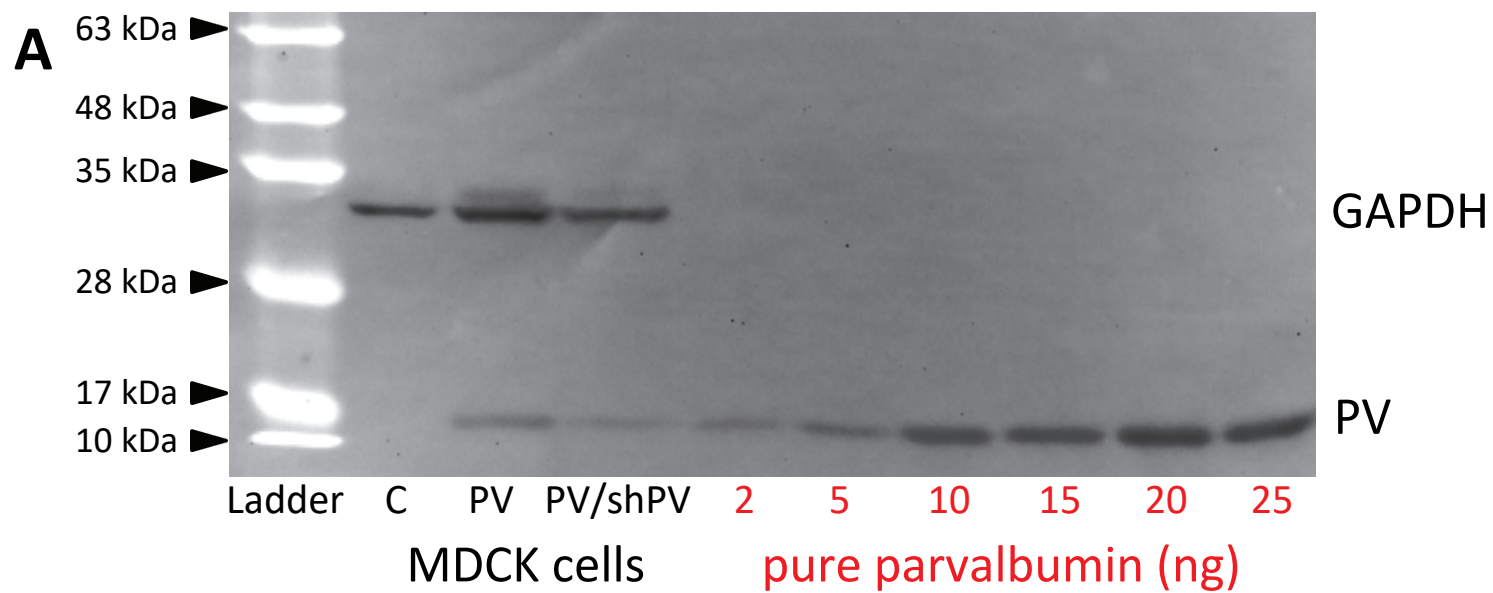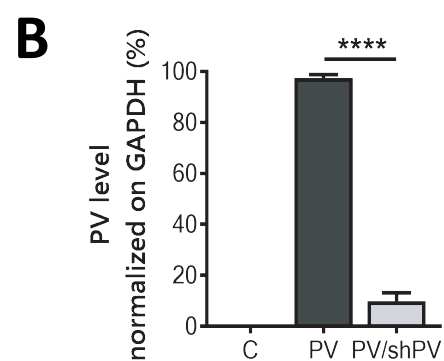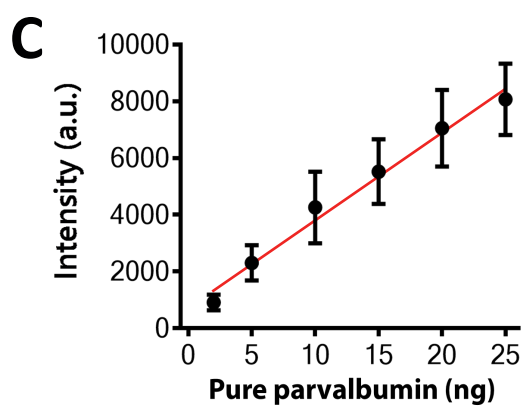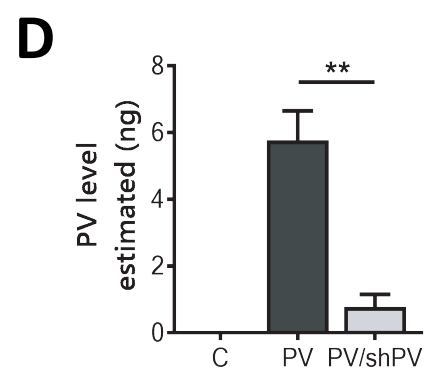

Supplement: Supplementary file 11 — Supplementary Fig. S1 Estimation of the PV concentration in MDCK cells. a) Detection of protein expression levels for PV (Mr:12 kDa) and GAPDH (Mr:35 kDa) in C-MDCK cells, PV-MDCK cells and PV/shPV-MDCK cells by Western blot analysis. Increasing amounts of purified PV (2, 5, 10, 15, 20, 25 ng) were used for PV determination in MDCK cells. b) Analysis of PV Western blot signals in MDCK cells. PV expression was below the threshold for detection in C-MDCK cells. A clear signal for PV was visible in PV-MDCK cells, as shown from a representative Western blot (a). PV expression of PV-MDCK cells was set as 100%, thus PV/shPV-cells expressed 10.43 ± 0.88% of PV protein compared to PV-MDCK cells. Determination of the quantity of PV per MDCK cell was estimated from the calibration curve showing increasing amounts of pure PV (c). According to the calibration curve, PV protein amounts in PV-overexpressing MDCK cells is equal to 5.77 ± 0.88 ng per cell and to 0.78 ± 0.38 ng in PV/shPV-MDCK cells (d) (PDF 400 kb) [file 18_2018_2921_MOESM11_ESM.pdf]

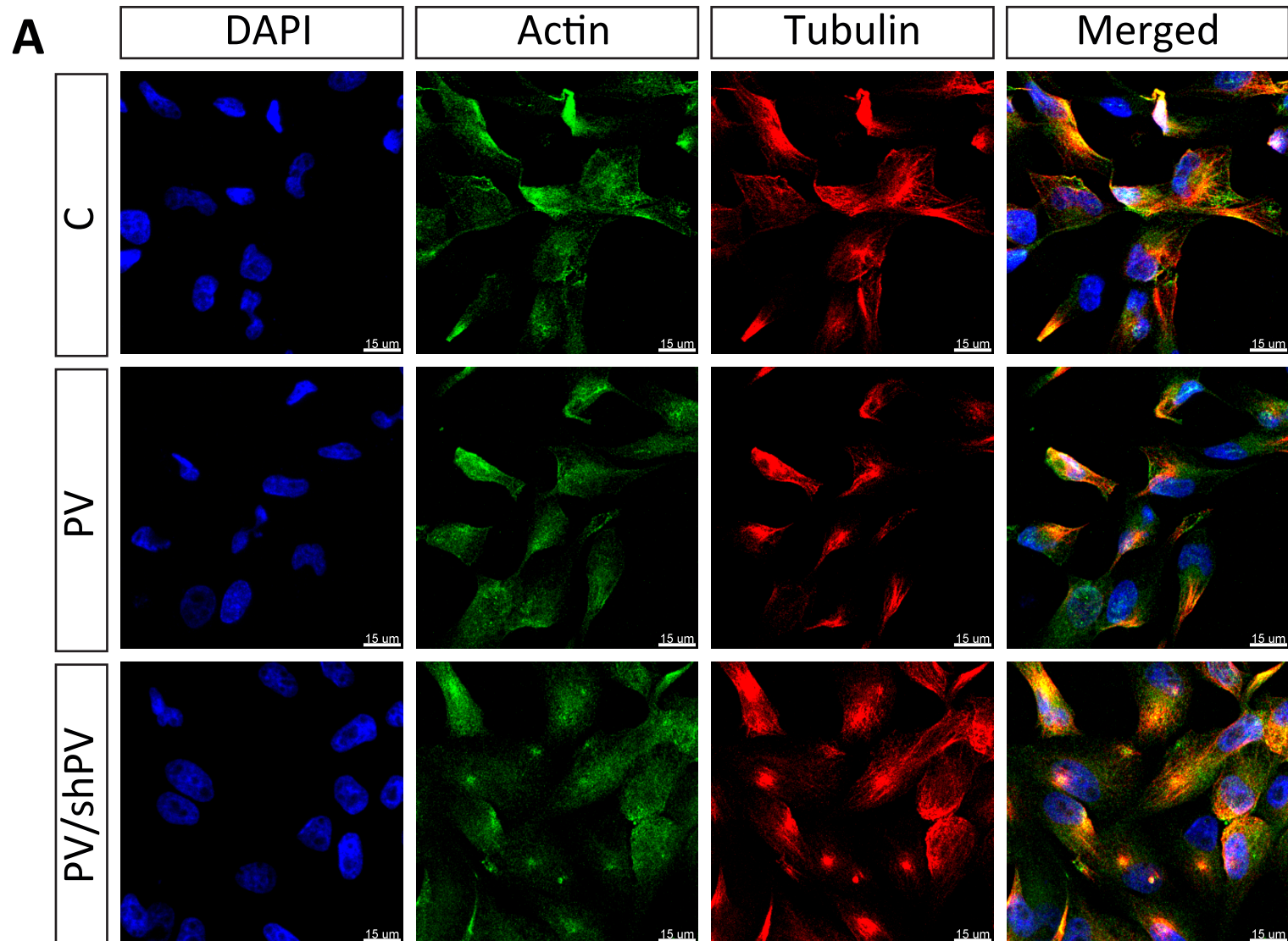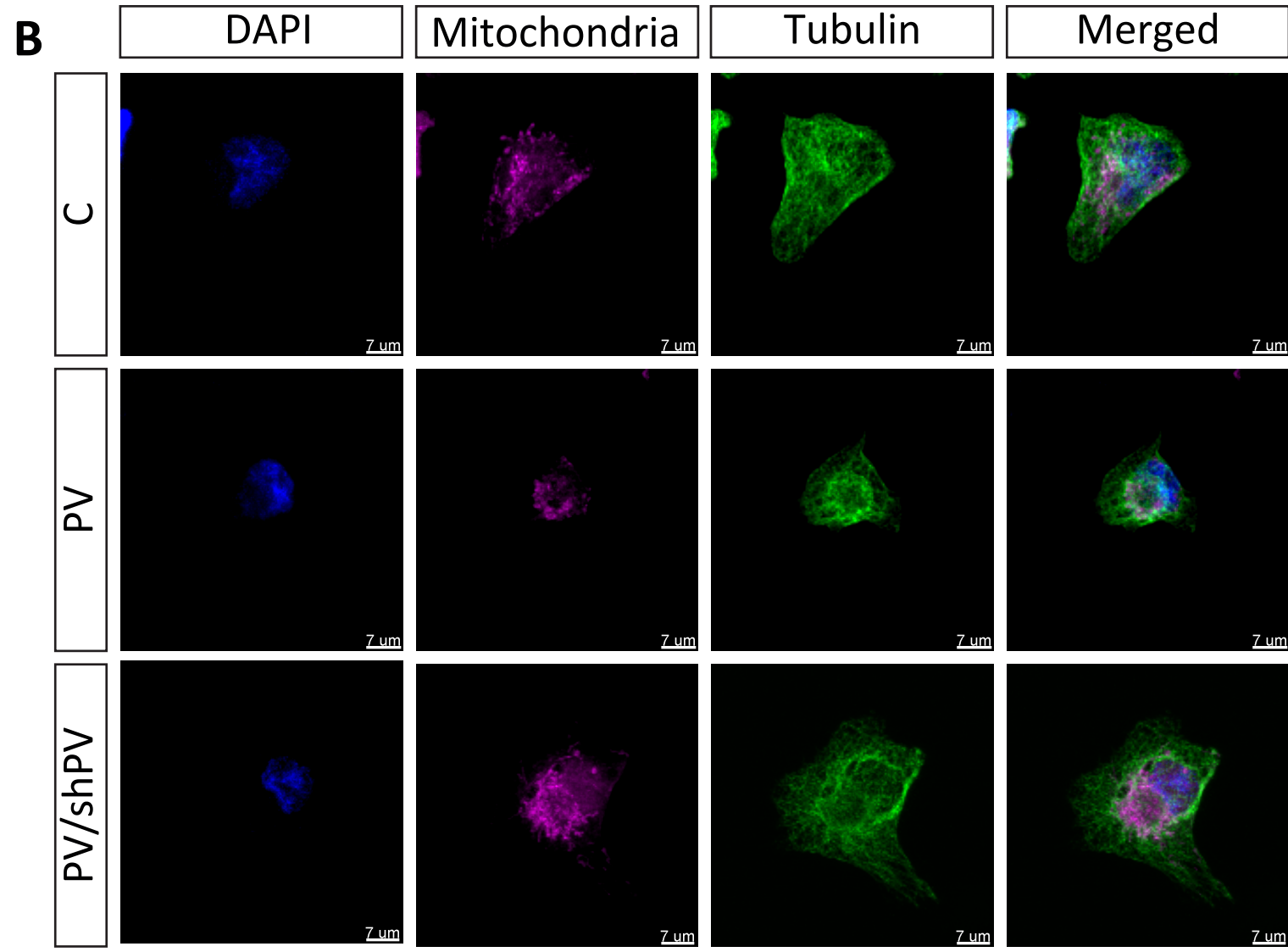

Supplement: Supplementary file 12 — Supplementary Fig. S2 Subcellular localization of actin and tubulin in MDCK cells. MDCK cells were plated for 24 h, fixed and stained for α-actin, α-tubulin and DAPI. a) Representative images show single Z-sections at the height of the largest diameter of the nucleus (DAPI, blue), actin (green) and tubulin (red) in fixed MDCK cells. b) MDCK cells were plated for 24 h, then loaded with MitoTrackerRed CMXRos, washed three times and then fixed and stained for α-tubulin and DAPI. Representative images of the nucleus (DAPI, blue), mitochondria (magenta) and tubulin (green) showed the organization of microtubules together with the distribution of mitochondria on microtubule tracks (PDF 9820 kb) [file 18_2018_2921_MOESM12_ESM.pdf]
